# Bulk and single-molecule analysis of a bacterial DNA2-like helicase–nuclease reveals a single-stranded DNA looping motor

**DOI:** 10.1093/nar/gkaa562

**Published:** 2020-07-04

**Authors:** Oliver J Wilkinson, Carolina Carrasco, Clara Aicart-Ramos, Fernando Moreno-Herrero, Mark S Dillingham

**Affiliations:** School of Biochemistry, Biomedical Sciences Building, University of Bristol, University Walk, Bristol BS8 1TD, UK; Department of Macromolecular Structures, Centro Nacional de Biotecnología, Consejo Superior de Investigaciones Científicas, 28049 Cantoblanco, Madrid, Spain; Department of Macromolecular Structures, Centro Nacional de Biotecnología, Consejo Superior de Investigaciones Científicas, 28049 Cantoblanco, Madrid, Spain; Department of Macromolecular Structures, Centro Nacional de Biotecnología, Consejo Superior de Investigaciones Científicas, 28049 Cantoblanco, Madrid, Spain; School of Biochemistry, Biomedical Sciences Building, University of Bristol, University Walk, Bristol BS8 1TD, UK

## Abstract

DNA2 is an essential enzyme involved in DNA replication and repair in eukaryotes. In a search for homologues of this protein, we identified and characterised *Geobacillus stearothermophilus* Bad, a bacterial DNA helicase–nuclease with similarity to human DNA2. We show that Bad contains an Fe-S cluster and identify four cysteine residues that are likely to co-ordinate the cluster by analogy to DNA2. The purified enzyme specifically recognises ss-dsDNA junctions and possesses ssDNA-dependent ATPase, ssDNA binding, ssDNA endonuclease, 5′ to 3′ ssDNA translocase and 5′ to 3′ helicase activity. Single molecule analysis reveals that Bad is a processive DNA motor capable of moving along DNA for distances of >4 kb at a rate of ∼200 bp per second at room temperature. Interestingly, as reported for the homologous human and yeast DNA2 proteins, the DNA unwinding activity of Bad is cryptic and can be unmasked by inactivating the intrinsic nuclease activity. Strikingly, our experiments show that the enzyme loops DNA while translocating, which is an emerging feature of processive DNA unwinding enzymes. The bacterial Bad enzymes will provide an excellent model system for understanding the biochemical properties of DNA2-like helicase–nucleases and DNA looping motor proteins in general.

## INTRODUCTION

DNA2 is an essential replication and repair factor found widely in eukaryotic proteomes (1). It is a multi-functional protein with roles in the processing of Okazaki fragments and stalled replication forks, and in the repair of double-stranded DNA breaks (2–6).The primary structure of DNA2 comprises an N-terminal RecB-family nuclease domain fused to a C-terminal SF1B helicase domain (7). In previous work, we hypothesised that the nuclease domain found in DNA2 and closely-related enzymes belonged to a new class of 4Fe–4S cluster associated domains (8), and this was later confirmed experimentally (9). We called these ‘iron-staple’ nuclease domains because of a unique arrangement of the cysteine residues that co-ordinate the cluster (8,10). Interestingly, we found that iron-staple domains were very commonly found associated with Superfamily I helicase domains, and further examples included components of some CRISPR systems (11). Given that DNA2 was thought to be restricted to eukaryotic and archaeal organisms, we were surprised to also find DNA2-like enzymes sporadically distributed in restricted niches of bacteria including *Geobacilli* and *Mycobacteria* (see Supplementary Figure S1 for further information).

From a mechanistic viewpoint, DNA2-like enzymes may be regarded as somewhat peculiar. Structural and biochemical analyses have shown that this domain arrangement places an endonuclease domain ‘in front’ of a translocating motor, such that the enzyme appears to have evolved to cleave the DNA track ahead of itself as it moves along DNA (12,13). In accordance with this proposition, the nuclease activity has been shown to be inhibitory to the translocase activity *in vitro* (12,14). This contrasts with the more intuitive and common domain arrangement seen in many other helicase–nuclease fusions, where the nuclease domain is transported behind the translocating motor, potentially leading to processive DNA degradation activity (15–17). To learn more about the activity of this intriguing class of proteins we have cloned, expressed and purified Bad, a bacterial DNA2-like enzyme from *Geobacillus stearothermophilus*. We show here that Bad contains an Fe–S cluster and identify the four cysteine residues that are likely to co-ordinate the co-factor. The purified enzyme possesses ssDNA binding, ssDNA-dependent ATPase, ssDNA endonuclease, 5′-to-3′ ssDNA translocase and 5′-to-3′ helicase activity. Single molecule analysis reveals that Bad acts as a fast and processive DNA looping motor, but that this activity is only evident under conditions in which the nuclease activity is suppressed, either by mutagenesis or by reducing the concentration of free Mg^2+^ ions in the reaction buffer. Thus, Bad displays highly similar biochemical properties to its homologues (including the human and yeast DNA2 proteins) and provides a robust model system for the study of DNA-looping helicases using single-molecule analysis.

## MATERIALS AND METHODS

### Identification, cloning and expression of Bad, a bacterial DNA2-like enzyme

Novel DNA2-like enzymes were identified by searching for uncharacterised proteins containing motifs characteristic of Superfamily I DNA helicases and Fe–S-containing nuclease domains (see Supplementary Figures S1–S3 for a comparison of Bad with the canonical DNA2 enzymes from yeast and human cells). A DNA2-like enzyme from *Geobacillus stearothermophilus 10* (DSM accession number 13240, Taxonomy ID: 272567) was cloned by PCR from genomic DNA using standard techniques. The untagged gene was ligated into the pET28a vector (Novagen) for expression using the T7 promoter system. The entire sequence of the cloned gene, which has been annotated as a AAA+ ATPase, was identical to that reported in the *G. stearothermophilus 10* genome (accession number WP_053413574). The gene encodes a 1245 amino acid protein with a molecular weight of 143 kDa and a theoretical extinction co-efficient of 195 960 M^−1^ cm^−1^. We note that a small number of other polypeptide sequences have been reported in the proteomes of *G. stearothermophilus* and closely related strains which are virtually identical to Bad but which feature an additional 25 amino acids at the N-terminus (e.g. accession number ADU93794.1). Given that sequences of this type are in a significant minority, and that the longer sequences all contain a methionine residue at position 26, it is possible that the start codon has been mis-assigned in these instances. We have also cloned and expressed an example of the putative longer Bad polypeptide, but this was found to be insoluble upon expression in *E. coli* (data not shown).

### Protein purification

Wild type and mutant Bad proteins were all overexpressed in *Escherichia coli* using the same method. pET28a-Bad was transformed into BL21(DE3) cells. Cells were grown in LB to mid-log phase before induction with IPTG (1 mM) for 16 h at 27°C. All buffers were degassed extensively prior to use in order to help prevent oxidation of the putative iron-sulphur cluster. The pellet (∼10 g) from 4 l of bacterial culture was resuspended in 30 ml lysis buffer (50 mM Tris–HCl pH 8.3, 1 mM EDTA, 150 mM NaCl, 10% glycerol, 5 mM DTT, 1 mM PMSF and Roche protease inhibitor cocktail) and sonicated on ice. After centrifugation at ∼50 000g for 30 min at 4°C, ammonium sulphate was added slowly with stirring to the cleared lysate to a final concentration of 50% (w/v) at 4°C. The precipitated protein was then recovered to a pellet by centrifugation at 50 000g for 30 min at 4°C. This pellet was then resuspended in Buffer A (20 mM Tris–HCl pH 8.0, 1 mM EDTA, 5% glycerol, 0.1 mM PMSF, 5 mM DTT, Roche protease inhibitor cocktail) up to a volume where the conductivity of the solution was 16 mSv, and then loaded at 2 ml/min onto a 5 ml Heparin column equilibrated in Buffer B (20 mM Tris–HCl pH 8.0, 1 mM EDTA, 100 mM NaCl, 5% glycerol, 0.1 mM PMSF, 5 mM DTT). After washing for 5 column volumes with Buffer B, a gradient was run over 20 column volumes into Buffer C (20 mM Tris–HCl pH 8.0, 1 mM EDTA, 1 M NaCl, 5% glycerol, 0.1 mM PMSF, 5 mM DTT). The Bad-containing fractions were pooled and diluted with Buffer A to give a final salt concentration of ∼170 mM NaCl before loading at 2 ml/min onto a 1 ml MonoQ column pre-equilibrated in Buffer D (20 mM Tris–HCl pH 8.0, 100 mM NaCl, 5 mM DTT). After washing with 5 column volumes Buffer D, the protein was eluted by running a gradient from Buffer D to 50% Buffer E (20 mM Tris–HCl pH 8.0, 1 M NaCl, 5 mM DTT) over 20CV. The most concentrated Bad-containing fractions were pooled and 1.5 ml was injected onto a pre-equilibrated Superdex 200 16/600 column in Buffer F (20 mM Tris–HCl pH 8.0, 300 mM NaCl, 5 mM DTT). The pool from the peak fractions was spin concentrated (Millipore, 50 kDa cut-off) to a final concentration of ∼15 μM. *Escherichia coli* SSB protein was expressed and purified as described previously (18) which is a modification of the method developed by Lohman (19).

### Iron chelation assay

The iron content of Bad preparations was determined using bathophenantroline, which chelates ferrous iron (Fe^2+^) resulting in the appearance of an absorbance peak at 535 nm, as described previously (8). Briefly, 10 μl of a 15 μM solution of Bad was mixed with 3 μl conc. HCl and incubated at 100°C for 15 min to denature the protein. After centrifugation at 13 000g for 15 min, the supernatant was removed and neutralised with 130 μl of 0.5 M Tris–HCl pH 8.5 and then treated with ascorbic acid to a final concentration of 0.26% to reduce the iron. After addition of bathophenanthroline disulphonic acid disodium salt to 0.021%, the samples were incubated for 1 h at room temperature and then the absorbance measured at 535 nm. The concentration of iron was calculated using the extinction coefficient 22 369 M^−1^ cm^−1^.

### ATPase assay

ATPase activity was measured by coupling the hydrolysis of ATP to the oxidation of NADH which gives a change in absorbance at 340 nm. Reactions were performed in a buffer containing 20 mM Tris–Cl pH 8.0, 50 mM NaCl, 5 mM DTT, 1 mM MgCl_2_, 50 U/ml lactate dehydrogenase, 50 U/ml pyruvate dehydrogenase, 1 mM PEP and 100 μg/ml NADH. Rates of ATP hydrolysis were measured over 1 min at 25°C. For calculation of *K*_DNA_ (defined as the concentration of DNA at which ATP hydrolysis is half-maximal), the ATP concentration was fixed at 2 mM. The Michaelis–Menten plot was performed at 20 μM DNA (nucleotide concentration) which is ∼10× the *K*_DNA_ value. The concentration of Bad was 10 nM in these assays unless indicated otherwise. The DNA substrate used in these assays was, unless stated otherwise, a 17mer ssDNA oligonucleotide of mixed sequence (ODN1; see Supplementary Methods for details).

### Nuclease assay

80 μM (nucleotides) ϕX174 Virion DNA in 50 mM Tris–HCl pH 7.5, 30 mM NaCl, 2 mM MgCl_2_, 2 mM DTT, 0.2 mg/ml BSA was treated with 1 μM Bad. 10 μl aliquots were quenched at time intervals over 60 min with an equal volume of stop buffer (100 mM EDTA, 1% SDS, 15% glycerol) and loaded onto a 1% agarose 1× TAE gel. The gels were stained with ethidium bromide and visualised by UV.

### Streptavidin displacement assay

Streptavidin displacement assays were based on the method of Morris and Raney (20), modified as in (21). 5 nM (molecules) of 5′-^32^P-labelled substrate oligonucleotides were incubated with 400 nM streptavidin in 25 mM HEPES pH 7.8, 25 mM NaCl, 2 mM MgCl_2_. Substrates were modified with either a 5′ or 3′ biotin moiety as indicated. The reaction was initiated by adding an equal volume of protein solution in the same buffer to give final concentrations of 10 nM Bad, 5 mM ATP and 8 μM biotin. The reaction was incubated at 37°C and stopped at certain points within a 4 min time course by quenching with an equal volume of stop buffer (300 mM EDTA, 400 mM NaCl, 30 μM poly(dT)). The products were separated on 10% polyacrylamide 1× TBE gels and visualised by phosphorimaging using a Typhoon imager. The sequences of the oligonucleotides used in this assay can be found in the Supplementary Methods.

### Helicase assay

Strand-displacement assays were based on the method of Matson (22), modified as in (21). 1 nM (molecules) of 5′-^32^P-labelled substrate oligonucleotides were incubated with 5 nM Bad in either 20 mM Tris–HCl pH 7.5, 2 mM MgCl_2_, 3 mM ATP, 1 mM DTT (which we refer to as the ‘low magnesium’ condition) or 20 mM Tris–HCl pH 7.5, 4 mM MgCl_2_, 2 mM ATP, 1 mM DTT (which we refer to as the ‘high magnesium’ condition) for 5 min at 25°C. The reaction was quenched at certain intervals over the time course by adding an equal volume of stop buffer (200 mM EDTA, 1% SDS, 10% (w/v) Ficoll 400 and 100 nM of an unlabelled form of the radiolabelled strand in the substrate to prevent re-annealing. The products were separated on 15% polyacrylamide 1× TBE gels and visualised by phosphorimaging using a Typhoon imager. The sequences of the oligonucleotides used in this assay can be found in the Supplementary Methods.

### Magnetic Tweezers translocation assay

We used a Magnetic Tweezers setup similar to one reported previously (23). Raw data was recorded at 60 Hz and filtered to 3 Hz for representation and analysis. Force values were calculated using the Brownian motion method applied to a DNA-tethered bead (24). The fluidic chamber was pre-incubated with 0.1 mg ml^−1^ of BSA proteins to minimize non-specific attachments of proteins and beads with the surface. DNA substrates (Figures 5 and 8) essentially consist of a DNA molecule of ∼6.6 kb containing a flap sequence (poly-dT oligo of 37 nt) in a specific-site, and flanked by two smaller fragments (∼1 or 0.6 kb) that act as the immobilisation handles as they are labeled with biotins or digoxigenins. The labeled parts are used to specifically bind each DNA end to a glass surface covered by anti-digoxigenins and to streptavidin coated magnetic beads. MT2 also contains a nick in a specific position within the top (DNA-nick top) or bottom (DNA-nick bottom) strand (Figure 8B). Doubly-tethered beads were identified by applying magnet rotation on the beads and not considered for the analysis.

Unless indicated otherwise, single-molecule translocation experiments were carried out at room temperature and at 8 or 14 pN as indicated, in a buffer that contained 20 mM Tris–HCl pH 8.0, 30 mM NaCl, 2 mM MgCl_2_, 5 mM DTT, 4 mM ATP and 100 μg ml^−1^ BSA (i.e. ‘low magnesium’ conditions) with Bad proteins at the quoted concentrations (30, 50 or 163 nM) using 1 μm bead (Dynabeads, Invitrogen). To initiate the reaction, Bad was flowed into the fluid chamber at 20 μl/min while the positions of the beads were measured by video microscopy. A fluidic chamber made with one parafilm layer (50 μl total volume) and vertical alignment magnets with a 0.11 mm gap were used to reach high applied forces. The quoted distances in base pairs were corrected using the value given by the worm-like chain model of rise per base pair of dsDNA at a given force. The unwinding rate was calculated by using the derivative of the smoothed data at 3 Hz in order to separate movement from pausing events (23). A detailed description of the construction of the magnetic tweezers DNA substrates can be found in the Supplementary Methods. DNA oligonucleotides used to construct tweezers substrates can be found in Supplementary Table S1. Sequences of the DNA fragments used in this work can be found in Supplementary Table S2.

## RESULTS

### Identification and purification of a bacterial DNA2-like enzyme

DNA2 is a DNA helicase–nuclease that is ubiquitous in eukaryotic cells and has been shown to be an essential DNA replication and repair factor (1). It is characterised by the fusion of a specific subtype of the RecB-family nuclease domain that contains a 4Fe-4S cluster (the ‘iron staple’ nuclease domain (8)) to a C-terminal SF1B helicase domain. In previous work, we identified and characterised the first example of an iron staple nuclease domain in the AddB subunit of the bacterial enzyme AddAB which, like DNA2, is implicated in the resection of double-stranded DNA breaks (8,25). Iron staple nuclease domains seem to be rare in nature but they are easily identifiable using a bioinformatics approach. In addition to four amino acid motifs associated with nuclease activity that are shared by all members of the RecB nuclease family, they also contain four strictly conserved Cys residues in a unique pattern that spans the entire domain (8,10). This arrangement results in the Fe–S cluster being critical for the overall structural integrity of the domain, at least in the case of AddAB. We used this bioinformatics signature to predict Fe–S nuclease domains in other proteins. Prominently, these included DNA2 and the Cas4 enzyme from CRISPR-Cas systems (9,11). Since DNA2 had been considered a eukaryotic protein, we were surprised to find that our searches also uncovered a few examples of bacterial and archaeal enzymes that displayed a DNA2-like domain architecture (Supplementary Figures S1–S3). Although these bacterial DNA2-like (Bad) proteins number relatively few and are found sporadically in the bacterial family tree, they are nevertheless broadly distributed. For example, DNA2-like proteins that are clearly homologous are found both in the Firmicute division of Gram-positive organisms and in some Gram-negative Proteobacteria (see Supplementary Figure S1). A second class of bacterial DNA2-like protein is also found in *Mycobacteria* and related organisms including *Rhodococcus*. Finally, similar proteins are also found in Euryarchaea including *Methanobacteria* (data not shown).

**Figure 1.**
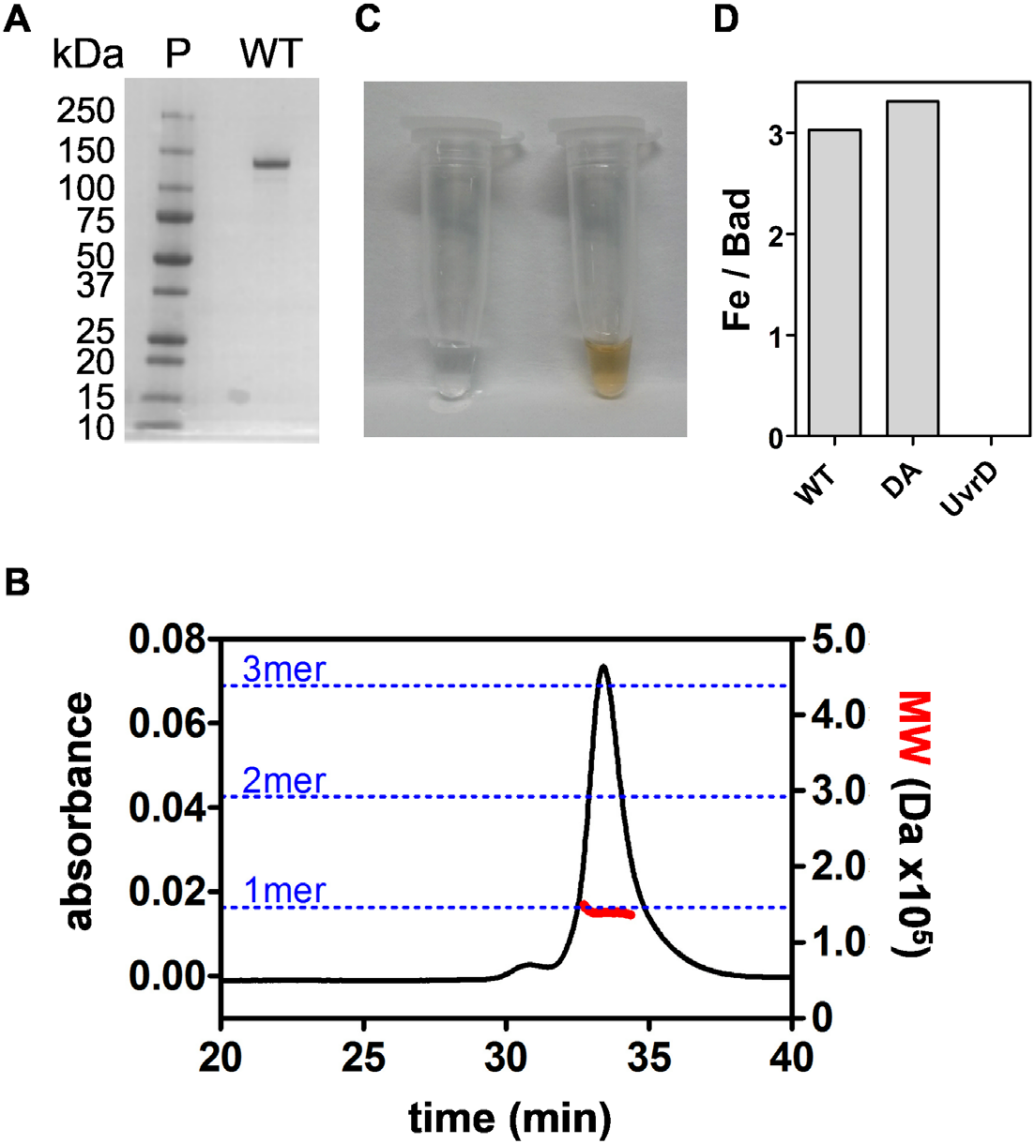
Purification of Bad; a DNA2 homologue from *Geobacillus stearothermophilus*. (**A**) SDS-PAGE gel showing purified wild type Bad used in this study. (**B**) SEC-MALS analysis of wild type Bad suggests that Bad is a monomer in free solution. (**C**) The concentrated (∼10 mg/ml) wild type Bad protein has a golden yellow colour which is characteristic of Fe–S proteins. Storage buffer is shown for comparison. (**D**) Bathophenanthroline assay reveals the presence of ∼3 mol of iron per mole of protein in Bad preparations. Data for wild type (WT) and nuclease-dead mutant (DA) Bad are shown, alongside a negative control experiment using *E. coli* UvrD helicase which does not possess an Fe–S cluster.

To investigate the properties of these enigmatic enzymes we cloned and purified the DNA2-like enzyme Bad from *Geobacillus stearothermophilus*. The protein was well expressed in *E. coli* and purified to near homogeneity without the use of tags (Figure 1A). SEC-MALS analysis of the wild type protein in the absence of ligands showed that Bad is a monomeric protein (Figure 1B). Purified Bad displayed a golden yellow colour, characteristic of Fe–S cluster containing proteins, and bathophenanthroline assays showed the presence of ∼3 mol of iron per mole of protein in the preparation (Figure 1C and D). Given that iron-sulphur clusters can be lost or converted between different structural classes during purification, and also that the primary structure of Bad is similar to the AddB nuclease domain (for which the Fe–S is well characterised), it is likely that the protein in fact contains a 4Fe-4S cluster (25). However, we cannot currently exclude the possibility that Bad contains a different class of Fe–S cluster. Mutation of the cysteine residues that are predicted to co-ordinate the Fe–S cluster (to alanine) resulted in the production of labile protein that was lost during purification (data not shown), presumably because of unfavourable effects on folding. In addition to these Cys to Ala mutations, we also altered amino acids in helicase motif I (i.e. the Walker A motif; K815A) and nuclease motif III (D150A) to generate proteins that would be expected to be devoid of ATPase and nuclease activity, respectively, for use in later experiments (26,27). The resulting mutants were well-expressed and purified to homogeneity in the same manner as the wild type (data not shown).

### Bad is a ssDNA-dependent ATPase and ATP-independent endonuclease

**Figure 2.**
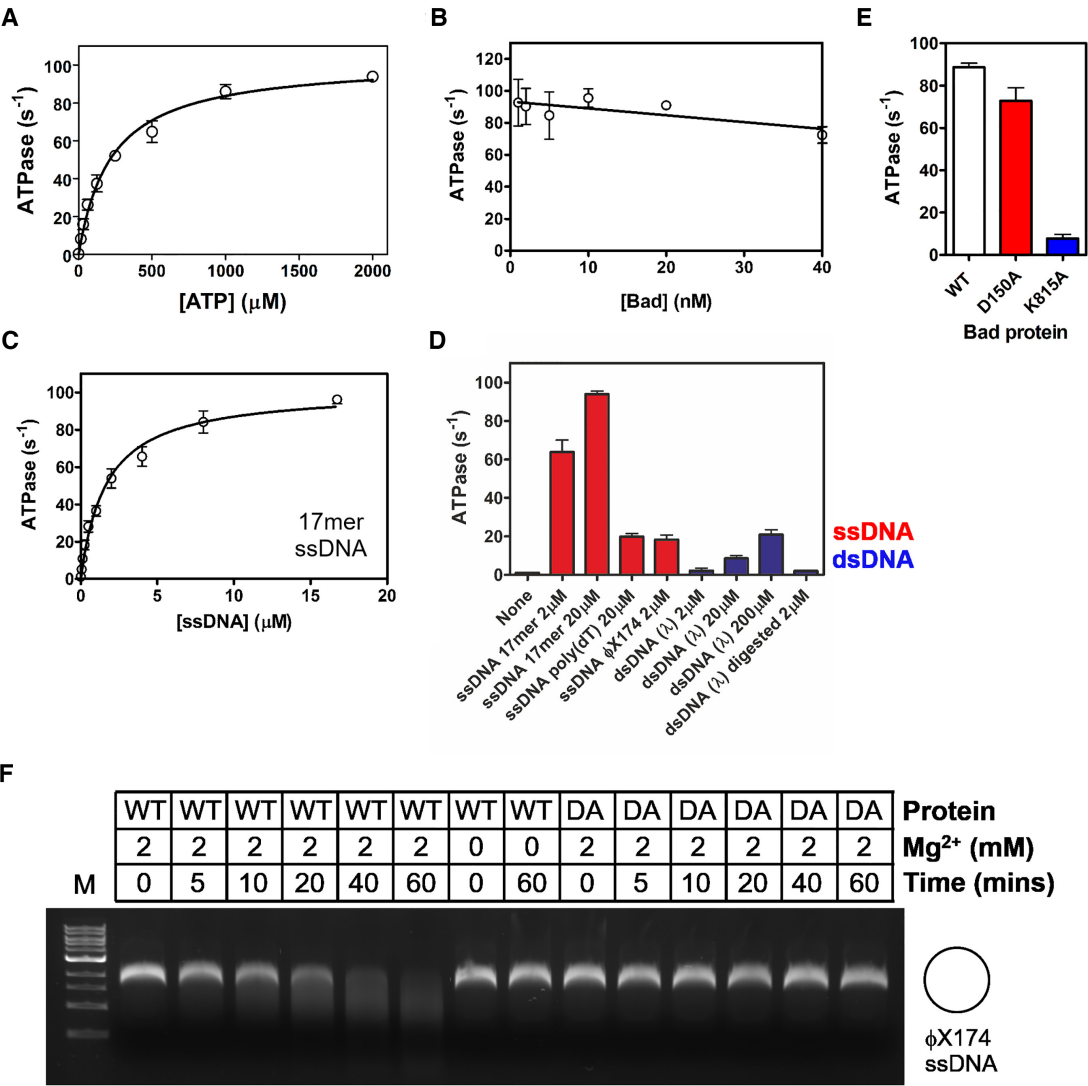
Bad is a ssDNA-dependent ATPase and ssDNA endonuclease. (**A**) Bad hydrolyses ATP with Michaelis–Menten kinetics (*K*_M_ = 223 ± 24 μM and *k*_cat_ = 101 ± 4 s^−1^). These measurements were made with Bad (10 nM) in the presence of saturating (20 μM) 17mer ssDNA oligonucleotide (ODN1, Supplementary Methods). (**B**) The ATP turnover rate as a function of Bad concentration provides no evidence for cooperativity in ATP hydrolysis. (**C**) Hydrolysis of ATP is ssDNA-dependent with half-maximal stimulation at *K*_DNA_ = 1.66 ± 0.22 μM. These measurements were made with Bad (10 nM) in the presence of saturating ATP (2 mM). (**D**) ATPase activity was measured with a variety of different nucleic acid co-factors. All single-stranded DNA molecules are effective activators of the ATPase activity, whereas dsDNA molecules are very poor co-factors. The presence or absence of DNA ends does not dramatically affect the ATPase rate. (**E**) Comparison of the ATPase activity of wild type Bad, nuclease-dead mutant (D150A) and helicase-dead mutant (K815A) at saturating ATP and DNA concentrations. (**F**) Single-stranded DNA endonuclease activity was assessed in the absence of ATP by incubating Bad (1 μM) with circular ssDNA as described in the Methods. In the presence of Mg^2+^ ions, wild type Bad completely degraded the DNA substrate, whereas the Bad D150A (DA) mutant showed no activity under the same conditions.

In the presence of saturating quantities of a 17mer ssDNA oligonucleotide (ODN1, Supplementary Methods), Bad hydrolysed ATP with Michaelis–Menten kinetics, yielding *k*_cat_ = 101 s^−1^ and *K*_m_ (ATP) = 220 μM (Figure 2A). The turnover number was unaffected by Bad concentration over a wide range of concentrations, providing no evidence for protein association affecting ATPase activity, and consistent with the idea that the protein is functional as a monomer (Figure 2B). In the absence of DNA, purified Bad displayed a basal ATPase turnover rate of ∼1 s^−1^ (Figure 2C and D). In the presence of saturating ATP, titrations with the 17mer ssDNA oligonucleotide revealed half maximal stimulation at *K*_DNA_ = 1.6 μM nucleotides (Figure 2C). Activation of the ATPase activity was most efficient with the short oligonucleotide substrate, which could suggest that free ends are particularly effective loading sites for Bad (Figure 2D). However, activation was apparent regardless of whether the ssDNA was linear (ssDNA 17 mer or poly(dT)) or circular (ssDNA ϕX174 Virion DNA), which is consistent with the nuclease activity that we also observed on circular ssDNA. In contrast, dsDNA was a relatively poor cofactor for ATPase stimulation. These properties are broadly typical of the Superfamily I helicases of which Bad is a member (27). Mutation of helicase motif I (K815A, also known as the Walker A motif) dramatically decreased the ATPase activity showing that it is intrinsic to the Bad polypeptide, whereas mutation of the nuclease motif (D150A) had little effect on the steady-state ATPase activity (Figure 2E). We next investigated the nuclease activity associated with Bad in the absence of ATP. To test this, we incubated Bad with circular ssDNA in the presence or absence of Mg^2+^ ions which would be expected to be required for activity. Bad was able to endonucleolytically cleave the ssDNA in a Mg^2+^-dependent fashion (Figure 2F). Mutation of nuclease motif III (Bad D150A) eliminated the observed DNA degradation, demonstrating that this activity is also intrinsic to the Bad polypeptide.

### Bad is a 5′-to-3′ DNA translocase and helicase

**Figure 3.**
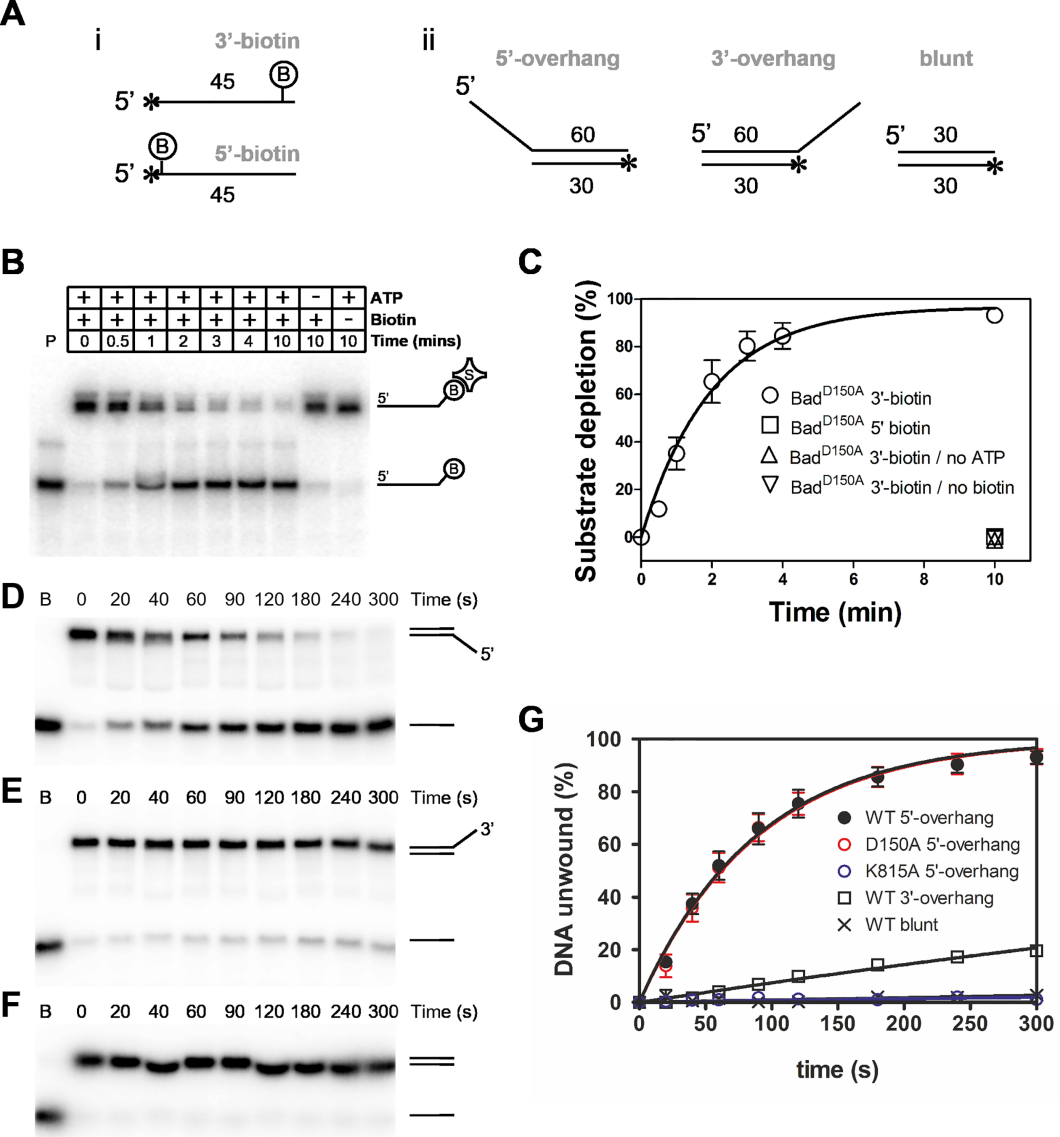
Bad is a 5′-to-3′ single-stranded DNA translocase and 5′-to-3′ DNA helicase. (**A**) Schematic representation of the DNA substrates used in (i) DNA translocation and (ii) DNA helicase assays. The names are in grey, the length of the component oligonucleotides are shown, the asterisks represent the position of 5′-radiolabelling, and ‘B’ shows the position of a biotin moiety. Sequences are available in the Supplementary Information. (**B**) Representative streptavidin displacement assay for Bad^D150A^ protein (10 nM) and an oligonucleotide labelled with biotin at the 3′ end (5 nM). (**C**) Quantification of the gel-based assays to compare the streptavidin displacement kinetics on 3′- and 5′-biotin labelled DNA. Control experiments on 3′-labelled DNA in the absence of either ATP or biotin are also shown. Bad D150A shows no 3′-5′ translocase activity. The data for the 3′-labelled DNA in the presence of both ATP and biotin were well fit to a single exponential to yield an apparent rate constant *k*_obs_ = 0.48 min^−1^. (**D**–**F**) Representative helicase assay gels for wild type Bad protein (5 nM) in low free Mg^2+^ ion conditions acting on duplex DNA substrates (1 nM) containing either 5′-terminated, 3′-terminated, or no ssDNA overhang as indicated by the cartoons. (**G**) Quantification of helicase gels for wild type protein with each of the three DNA substrates performed in low free Mg^2+^ ion conditions (see Methods for details). Data for Bad^D150A^ and Bad^K815A^ are also quantified for the 5′-terminated ssDNA overhang substrate only to allow comparison with wild type. Where appropriate the data were fit to a single exponential rise to yield apparent rate constants for unwinding (WT 5′-substrate = 0.66 min^−1^, D150A 5′-substrate = 0.66 min^−1^, WT 3′-substrate = 0.05 min^−1^).

To characterise the anticipated DNA motor activity of Bad, we first employed classical translocase and helicase assays to establish the existence and polarity of any such activity (27) (Figure 3A). We initially used the nuclease mutant (D150A) and ‘low free magnesium’ conditions (see the Methods) in order to avoid complications associated with degradation of the DNA substrates. Single-stranded DNA translocation was monitored using a streptavidin displacement assay (20). In this assay, oligonucleotides are labelled with biotin at either the 5′ or the 3′ end, and then bound to streptavidin. Translocating motor proteins are typically able to displace the streptavidin in an ATP-dependent manner, but only if they translocate towards the target biotin moiety, and this can be monitored as the loss of a gel shift using native gel electrophoresis. The Bad D150A mutant protein was able to efficiently displace streptavidin from the ends of 3′-biotinylated oligonucleotides, and this activity was completely dependent on ATP and free biotin (Figure 3B, C). The free biotin acts to trap displaced streptavidin and prevent re-binding to the oligonucleotide. Therefore, the ‘no biotin’ experiment serves as a control to show that the streptavidin has been displaced from the oligonucleotide rather than having been cleaved from the oligonucleotide by nuclease activity. In contrast, the Bad D150A protein showed no detectable streptavidin displacement activity on 5′-biotinylated oligonucleotides (Figure 3C). These data suggest that Bad is a 5′-to-3′ ssDNA motor protein. DNA unwinding activity was monitored using classical strand displacement helicase assays for both wild type and mutant Bad proteins under low free magnesium conditions (22). These assays determine DNA unwinding polarity by comparing activity on three test substrates, two of which comprise short DNA duplexes flanked by either a 5′- or a 3′-ssDNA overhang, and one of which contains an equivalent duplex with no overhang. Bad was only able to efficiently unwind duplexes flanked by 5′-terminated ssDNA overhangs (Figure 3D–G). This data is consistent with the translocase assays, shows that Bad displays 5′-to-3′ polarity, and classifies the enzyme as a SF1B helicase (28).

### Bad displays coupled helicase and nuclease activities

**Figure 4.**
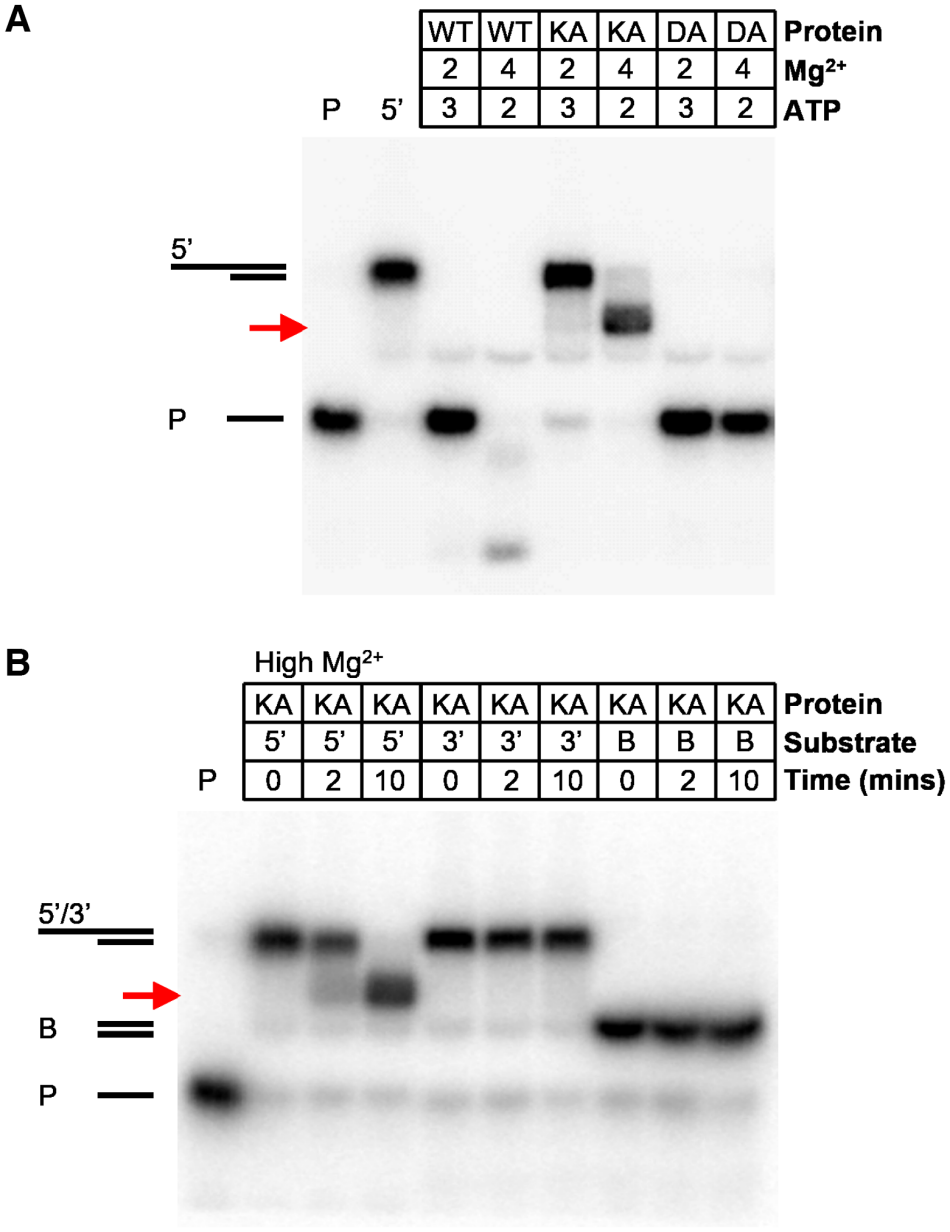
Bad displays coupled helicase and nuclease activities and binds in a well-defined orientation to 5′-overhang DNA substrates. (**A**) Helicase assays were performed with wild type or mutant Bad protein (5 nM) and 5′-terminated DNA substrates (1 nM) in either low or high free Mg^2+^ ion conditions. (**B**) Helicase assays were performed with the helicase-dead (K815A) mutant (5 nM) on 5′-terminated overhang, 3′-terminated overhang or blunt DNA substrates (1 nM). The substrates used are shown in schematic form in Figure 3A. In both panels, the red arrow indicates the position of an endonucleolytic cleavage product formed uniquely by the ATPase-dead mutant on the 5′-overhang substrate. Lanes containing the substrate only (5′) and the free short ssDNA product of unwinding (P) are labelled.

We next analysed the activity of Bad under high free Mg^2+^ conditions (see the Methods) which promote both helicase and nuclease activity. This resulted in the formation of different and more complex unwinding products (Figure 4). For the junction with a 5′-terminated ssDNA overhang, wild type Bad both unwound and degraded the labelled DNA strand whereas the nuclease-dead mutant (D150A) only unwound it (Figure 4A). Interestingly, the helicase-dead mutant (K815A) produced a highly specific-cleavage product, suggesting that it binds to this 5′-overhang substrate in a preferred orientation that leads to precise endonucleolytic cleavage when ATP hydrolysis cannot take place. No such product was formed with this mutant protein on DNA molecules containing a 3′-ssDNA overhang or with blunt ends (Figure 4B). Using mass spectrometry, the position of this endonucleolytic cleavage event was mapped to a position on the 5′-overhang that was 13 nucleotides from the ss-ds junction (Supplementary Figure S4). Experiments with DNA junctions containing different duplex and 5′-overhang lengths showed that this cleavage position was always 13 nucleotides away from the ss–ds junction rather than being measured relative to the free 5′-end (Supplementary Figure S4). These data suggest that Bad somehow specifically recognises the ss–dsDNA junction within a 5′-overhang substrate. In further support of this idea, incubation of a ssDNA-only substrate with wild type Bad leads to less well-defined products and a more complete degradation of substrate, and the helicase-dead mutant displays negligible activity on this substrate (Supplementary Figure S4). Therefore, 5′-overhangs apparently act as efficient loading sites for Bad and can help position the endonuclease domain, but the enzyme's cleavage positions are also dependent on the active DNA motor to which the endonuclease is physically coupled.

### Single molecule analysis of Bad reveals a fast and processive DNA motor

**Figure 5.**
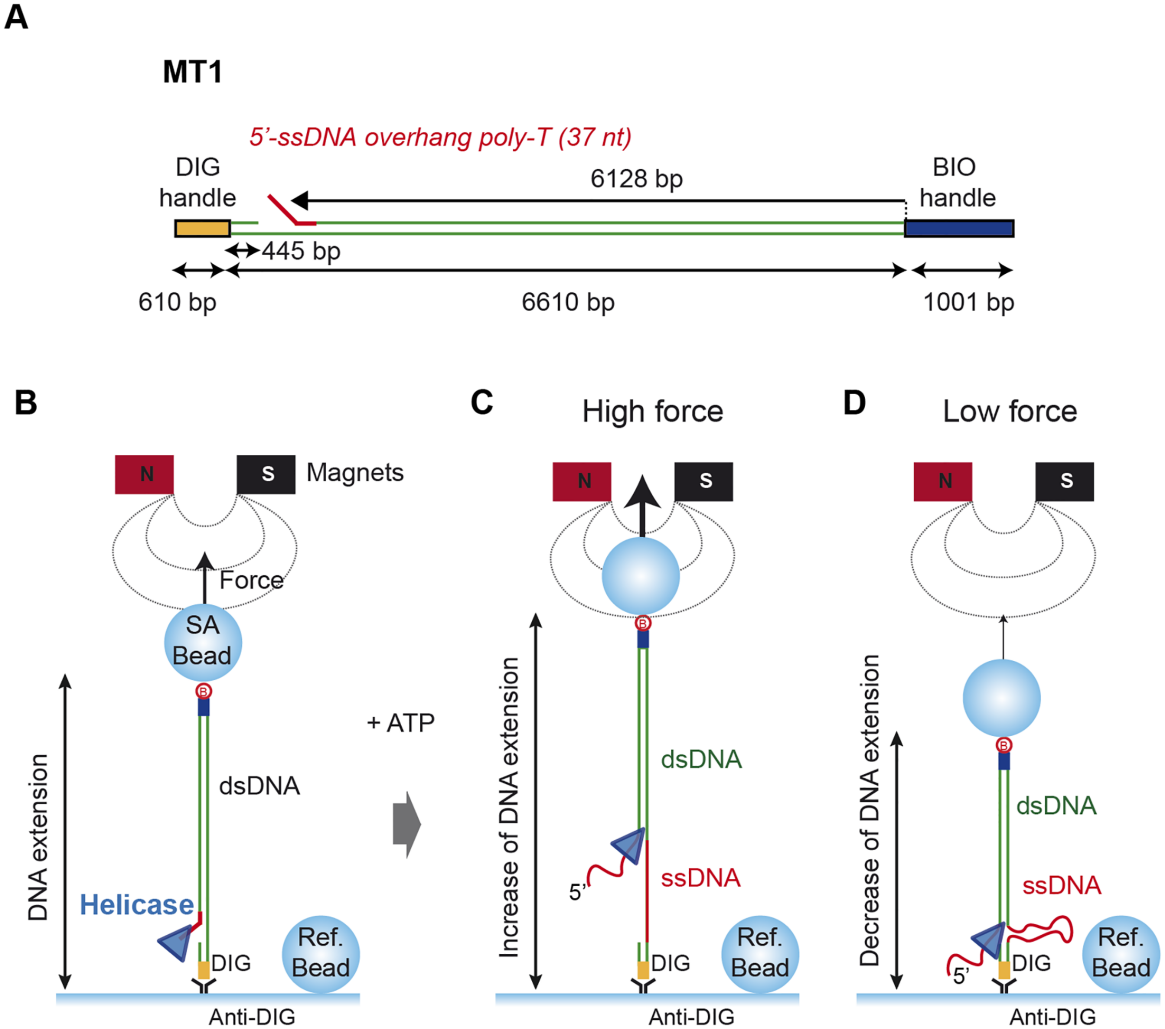
Magnetic Tweezers assay for monitoring DNA unwinding by Bad. (**A**) Schematic representation of the MT1 DNA substrate employed for the single molecule unwinding assay. It contains two handles labelled with biotins and digoxigenins which bind to the magnetic bead and glass surface, respectively. The insert (∼6.6 kb) contains a 5′-ssDNA overhang at ∼6.1 kb from the biotinylated DNA end opposing a short ssDNA gap of 37 ntds. (**B**) In the experimental set-up, tethered DNA molecules are incubated with Bad proteins in the flow cell and Bad is loaded onto the 5′-ssDNA overhang. Note that, when a protein displays canonical NA unwinding (helicase) activity, the bead is expected to either (**C**) increase in height if the restraining force on the bead is high (i.e. ≥6 pN, because ssDNA is longer than dsDNA under these conditions) or (**D**) decrease in height under a low force regime (i.e. ≤6 pN, in which ssDNA is compacted compared to duplex).

The ability of bulk helicase assays to provide mechanistic insight into DNA translocation and unwinding reactions is limited. This is because the observed activity is a measure of not only the DNA translocation and unwinding, but also of association/dissociation of the helicase and any failed unwinding events caused by lack of processivity or duplex re-annealing. These complications can be side-stepped by employing single molecule techniques in which translocation and unwinding are either directly observed or inferred from changes in the mechanical properties of the substrate DNA (29). Therefore, we used a magnetic tweezers (MT) approach to monitor the dynamics of DNA unwinding by Bad (Figure 5). We designed a ∼6.6kbp DNA substrate containing a free 5′-terminated poly-T ssDNA (37 nt) to act as a loading site for the enzyme located 445 bp from one DNA end (MT1 substrate, Figure 5A). DNA substrates were attached at one end to the bottom glass surface of a flow cell and at the other end to paramagnetic beads. External magnets were used to apply force in order to extend and/or twist the DNA, while the Z height of the bead was monitored (Figure 5B). DNA unwinding can be monitored in this set-up because single- and double-stranded DNA display different force extension curves (30). In high applied-force (*F* ≥ 6 pN) regimes, ssDNA is longer than duplex DNA and so helicase activity leads to an increase in the Z position of the bead (Figure 5C). Under low forces (*F* < 6 pN), single-stranded DNA is shorter than duplex and unwinding leads to a reduction in the height of the bead (31–33) (Figure 5D).

**Figure 6.**
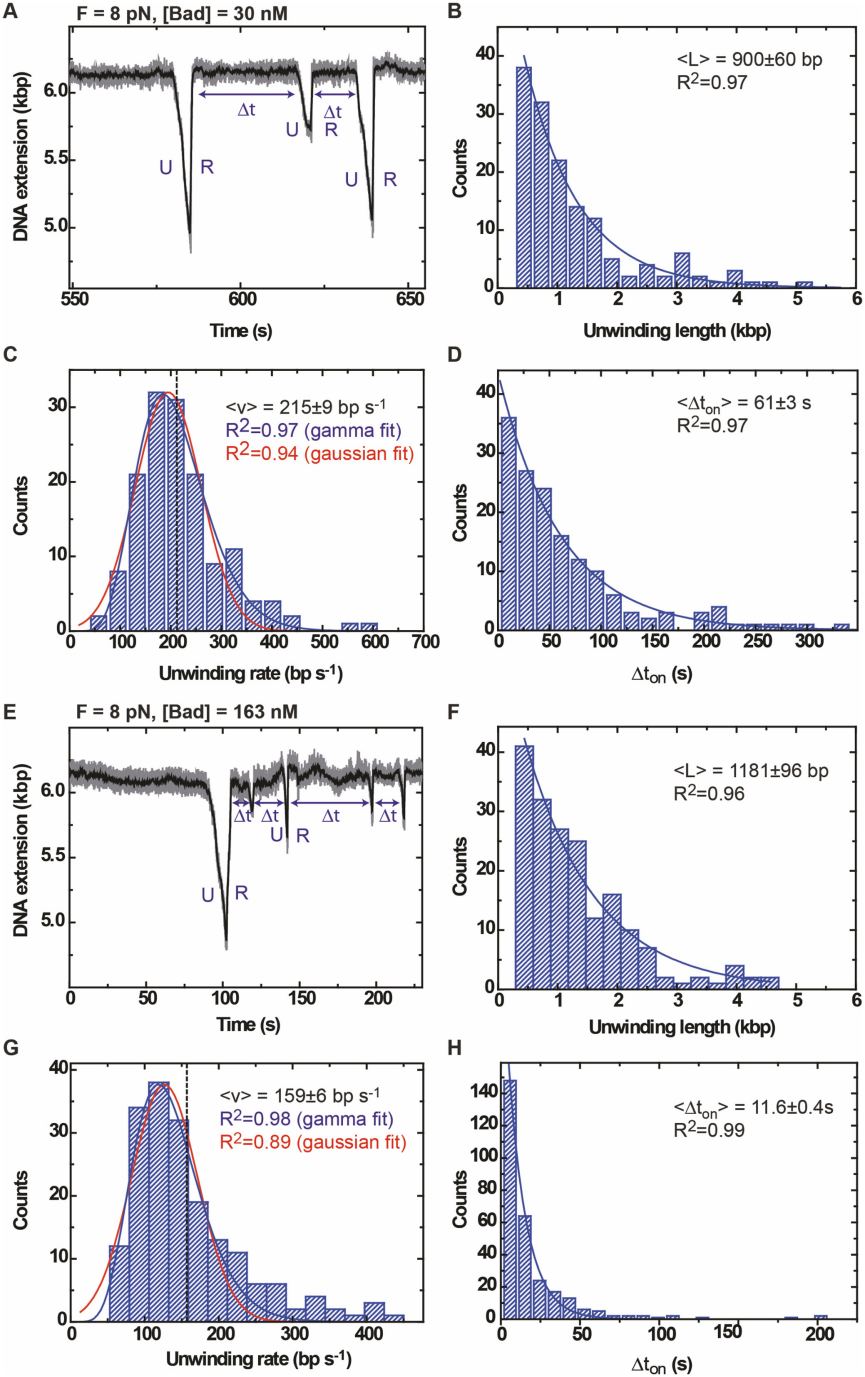
Dynamics of DNA unwinding by Bad at low and high protein concentrations. (**A**) Typical unwinding trace measured at 30 nM Bad^D150A^ concentration and 8 pN force shows unwinding (U) and rapid rehybridization (R) events. We observe a decrease in DNA extension for the unwinding phase followed by a rapid increase in DNA extension when the enzyme detaches from DNA allowing a rehybridization and recovery of the initial DNA extension. (**B**) The distribution of the unwinding length events decays exponentially governed by the mean unwinding length event <*L*> = 900 ± 60 bp (error of fitting, *n* = 147). (**C**) The distribution of the unwinding rate events was fitted with a Gamma function (blue) and with a Gaussian function (red). The quality of the Gamma and Gaussian fits are quite similar (see R^2^ values). The dashed line indicates the mean value of the rate. In total 57 tethered beads were analysed showing a total of 147 unwinding events. (**D**) Distribution of the dwell time between events Δ*t* decays exponentially governed by the mean time <*Δt*> = 61 ± 3 s (error of fitting, *n* = 147). (**E**) Typical unwinding curve at 163 nM Bad^D150A^ and 8 pN force shows several unwinding (U) and quick rehybridization (R) events. (**F**) Distribution of the unwinding length events decays exponentially governed by the mean unwinding length event <*L*> = 1181 ± 96 bp (error of fitting, *n* = 184). (**G**) The distribution of the unwinding rates was fitted with a Gamma function (blue) and with a Gaussian function (red). The dashed line indicates the mean value of the rate. In total, 184 unwinding events from 92 tethered beads were analysed. (**H**) Distribution of the dwell time between events *Δt* decays exponentially governed by the mean time <Δ*t*> = 11.6 ± 0.4 s (error of fitting, *n* = 290).

In our initial experiments, it became apparent that neither an intact duplex, nor a duplex containing a site-specific nick were unwound by wild type or mutant Bad (Supplementary Figure S5A). This was unsurprising, given that our bulk helicase assays had suggested that fully duplex DNA was a poor substrate, and that Bad bound to 5′-overhang substrates in a preferred orientation. Therefore, tethered MT1-DNA-magnetic beads were incubated with Bad^D150A^ (the nuclease-dead mutant) at 8 pN applied force in the presence or absence of ATP. At all concentrations of Bad^D150A^ tested (30, 50 and 163 nM), ATP-dependent helicase activity was observed as many cycles of unwinding (U) and rehybridization (R) in both high and low free Mg^2+^ ion conditions (a representative trace is shown in Figure 6A). In contrast, unwinding by the wild type enzyme was only observed under conditions of low free Mg^2+^ ions and high ATP which suppress the nuclease activity (Supplementary Figure S5B). This is consistent with the bulk data presented above and suggests that the nuclease activity of the wild type enzyme inhibits its own helicase activity, presumably either by efficiently cleaving the 5′-terminated loading strand from the substrate and/or by cutting the DNA track ahead of the SF1B motor domain. Additional control experiments without ATP or with the Bad^K815A^ (helicase-dead mutant) did not show any activity (Supplementary Figure S5C). All of the further experiments described below use the nuclease mutant in low free Mg^2+^ conditions to minimize nuclease activity.

At a relatively low (30 nM) concentration of Bad^D150A^, the observed unwinding length was exponentially-distributed, as is expected under standard models for helicase processivity (34), with an average distance travelled of 900 ± 60 bp (error of fitting, *n* = 147) (Figure 6B). The translocation rate distribution was well-fit to a Gaussian function with a mean value of 215 ± 9 bp s^−1^ (SEM, *n* = 147) (Figure 6C). Finally, the dwell time between unwinding events (Δ*t*) was exponentially-distributed with a time constant of 61 ± 3 s (error of fitting, *n* = 152) (Figure 6D). Pausing, ‘backsliding’ (events in which unwinding restarts after a fast but partial rehybridization) and changes of rate during unwinding were rare under these conditions (see Table 1 and Supplementary Figure S6 for quantification and examples of such events). We do not detect ‘strand-switching’; a commonly observed phenomenon in single molecule helicase assays in which the translocating enzyme abruptly changes direction (see (30) for examples). We also characterised the DNA unwinding activity of Bad^D150A^ at a higher fixed concentration (163 nM) and similar unwinding and rehybridization events were observed (Figure 6E). However, although the frequency of backsliding remained similar, pauses during both unwinding and rehybridization now occurred more frequently (Table 1). From a total of 184 events, 21% and 37% of traces showed pauses in the unwinding and rehybridization respectively. The observed lengths of unwinding events were exponentially distributed with an average distance travelled of 1181 ± 96 bp (error of fitting, *n* = 184) (Figure 6F). However, the rates of the unwinding events were now poorly described by a gaussian distribution. Instead, fitting to a gamma function showed an average value of 159 ± 6 bp s^−1^ (SEM, *n* = 184) (Figure 6G). The distribution of the dwell times ($\Delta t$) between two unwinding events observed on the same DNA molecule was exponentially distributed with a shorter time constant of 11.6 ± 0.4 s (error of fitting, *n* = 290) (Figure 6H).

**Table 1.** Frequency of Bad^D150A^ pauses and backsliding during DNA unwinding and rehybridization

	30 nM Bad	50 nM Bad	163 nM Bad
**#beads, #events**	57, 147	43, 106	92, 184
**Pauses during unwinding events**	5%	10%	21%
**Pauses during rehybridization events**	22%	41%	37%
**Backsliding during unwinding events**	24% (of which 97% complete)	21% (of which 52% complete)	27% (of which 58% complete)

Higher Bad concentrations result in more pauses during unwinding and rehybridization. The frequency with which backsliding events are observed is approximately constant, but a greater proportion of backsliding events lead to complete rehybridization if the Bad concentration is low.

**Figure 7.**
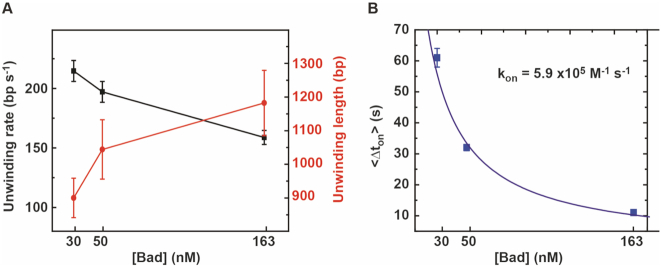
Effect of Bad concentration on DNA unwinding parameters. (**A**) Unwinding rate and unwinding length at different Bad^D150A^ concentrations (30 nM, 50 nM, 163 nM) and 8 pN force. The mean value and standard error of the mean are shown for different protein concentrations (30, 50, 163 nM). (**B**) Plot of the mean dwell time <*Δt*> versus Bad protein concentrations. Data were fitted with a simple hyperbolic function to obtain the binding rate constant. The parameter Δt is defined as the time between the end of an unwinding event and the next initiation event at *fully hybridised* DNA molecules (which are assumed to occur because of association of a Bad molecule from free solution). Initiation events following backsliding (which are assumed to be caused by a pre-bound Bad) are therefore excluded from the calculation, as would be appropriate in calculating the rate constant for Bad-DNA association.

In summary, we found that the apparent translocation rate decreases at higher Bad concentrations, whereas the initiation frequency, the pause frequency and processivity all increase (Figure 7A and Table 1). These observations can all be explained by the idea that many more initiation events occur at high concentrations of Bad, such that multiple Bad molecules may be translocating on a single substrate at the same time. This could hinder DNA translocation, leading to pausing, spontaneous changes in the rate of unwinding and a complex rate distribution as observed, but might also improve the processivity by disfavouring re-hybridisation. The constant frequency of the backsliding events (Table 1; Supplementary Figure S6D) regardless of protein concentration suggests that these are an intrinsic property of the functional form of the enzyme and are the result of re-initiation of unwinding by the same enzyme, rather than an artefact caused by rebinding of enzyme from free solution or overloading of the DNA substrate with multiple Bad proteins. We hypothesize that backsliding results from dissociation of the DNA motor domains of Bad from the DNA track, but that the protein can retain a loose grip on the substrate (probably with the non-translocated strand for reasons discussed below), allowing it to re-engage and resume translocation. The relationship between the dwell time $\Delta {t_{on}}$ and [Bad] suggests that initiation and re-initiation events from the loading site are caused by binding of Bad from free solution and allow us to calculate the second order rate constant for this process (defined as ${k_{on}} = {\rm{\ }}1{\rm{\ }}/{\rm{\ }}( {\langle {\Delta {t_{on}}} \rangle *[ {Bad} ]} )$ as ${k_{on}}$ = 5.9 × 10^5^ M^−1^ s^−1^ (Figure 7B).

### DNA unwinding by Bad is coupled to single-stranded DNA loop extrusion

An interesting and somewhat unexpected feature of these traces is that the activity of Bad manifests itself as an ATP-dependent decrease in the bead height despite the high (8 pN) restraining force. At this applied force, the ssDNA product is actually expected to be longer than the duplex substrate (30) (Figure 5B and C). Indeed, even at a restraining force of 14 pN, we found that Bad caused the bead height to processively *decrease*, and the traces were very similar to those measured at 8 pN (Supplementary Figure S7). To confirm that we were indeed observing DNA strand separation, we also performed experiments in the presence of bacterial single-stranded DNA binding (SSB) protein. We reasoned that, if ssDNA is formed during ATP-dependent translocation, then DNA rehybridization events (marked R) should be much slower in the presence of SSB. In these experiments, tethered DNA molecules were incubated with both Bad^D150A^ and SSB proteins at 8 pN applied force (Supplementary Figure S8). Under these conditions, although rehybridization did still occur, the overwhelming majority (93%) of the events showed a dramatically slower rehybridization. Moreover, rehybridization could be completely eliminated at higher [SSB]. Interestingly, ‘*backslide*’ events were also substantially reduced (to ∼10% of the total events analyzed) in the presence of SSB.

**Figure 8.**
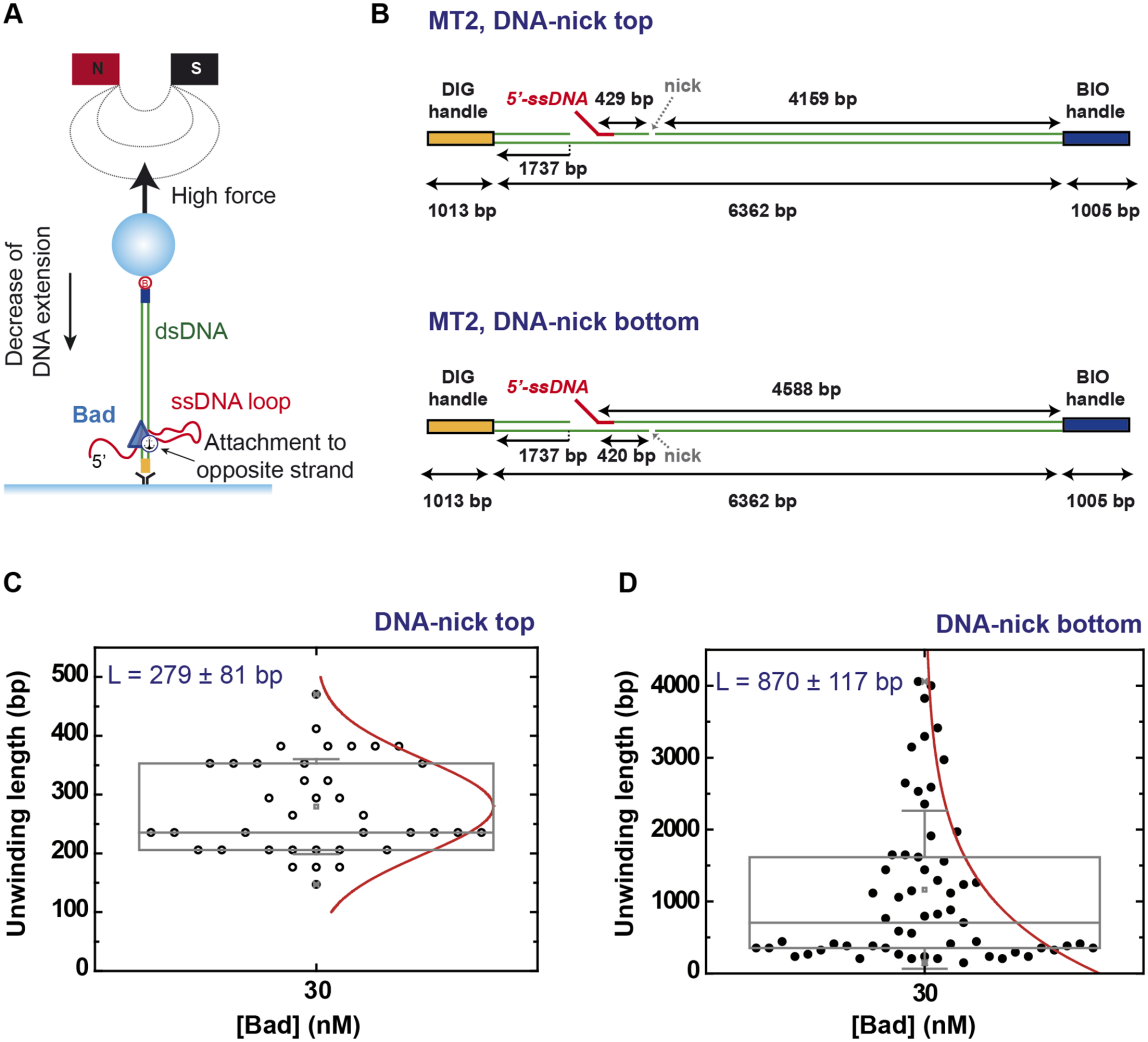
Model for Bad-dependent single-stranded DNA loop extrusion and unwinding activities using nicked DNA substrates. (**A**) The decrease in bead height we observe due to ATP-dependent Bad translocation and unwinding can be explained by a model in which the non-translocated (opposite) strand forms a ssDNA loop that is extruded from the enzyme complex during movement. If the enzyme does *not* retain a contact with the DNA behind the flap on the translocated strand, then there is no topological constraint to movement and the decrease in bead height will simply be equivalent to the distance moved forward by the enzyme. This idea can be tested using nicked DNA substrates. (**B**) Cartoons of the nicked DNA substrates used. (**C**) Box plot graphs of the observed unwinding length for DNA with a nick in the top (translocating) strand and for (**D**) DNA with a nick in the bottom strand. Experiments were performed at 30 nM Bad concentration with the nuclease mutant. Box plots indicate the median, 25th and 75th percentiles of the distributions and the whiskers show the standard deviation. For both data sets, the red line shows the distribution of the data which is gaussian for the top strand nick but exponential for the bottom strand nick.

We considered several possible models for how Bad activity might decrease the height of the bead. Firstly, the bead height change could be caused by an experimental artefact, such as the Bad protein sticking to the flow cell surface during translocation. This is unlikely because the observed enzyme activity requires the 5′-ssDNA loading site, this is located at a position distant from the glass surface (∼445 bp), and the traces provide no evidence to suggest that binding of Bad causing the loading site to interact with the glass surface. This is true even when the loading site is re-positioned further from the surface (for example in the nicked substrates that will be discussed further below; Figure 8B). Therefore, we favour an alternative possibility in which the Bad monomer contains multiple DNA binding sites and remains bound to the non-translocating strand near the loading region of the substrate while translocating on the 5′-strand (Figure 8A). In this scenario, movement along the DNA would cause looping on the non-translocating strand, leading to the formation of a ssDNA loop and the observed decrease in bead height.

### The arrest of Bad at single-strand nicks confirms the loop extrusion model

In a model where Bad remains bound to parts of the DNA substrate other than the translocating strand, the reduction in bead height associated with DNA translocation may either be explained by simple loop extrusion as we propose, or by the introduction of positive writhe in the DNA ahead of the translocating motor. In the first scenario, the decrease in bead height would be directly equivalent to the distance travelled into duplex DNA by Bad. In the second scenario, the interpretation of the relationship between translocation rate (in base pairs) and observed bead height (in microns) would be complex, with relatively small translocation events causing larger effects on the beads. To formally discriminate between such models and to test how Bad translocation was affected by damage to either strand of the duplex, we next performed experiments with substrates containing nicks. The substrate DNA-nick-top contains a nick in the 5′-to-3′ translocated strand, whereas DNA-nick-bottom is nicked in the 3′-to-5′ non-translocated strand (Figure 8B). We reasoned that if translocation ceased at a distance equivalent to the distance between the loading site and the lesion, then we were observing simple loop extrusion. Initial control experiments using nicked DNA molecules (Supplementary Figure S5A) or even a 63 base-gap (Supplementary Figure S5D) without a 5′-ssDNA overhang as a loading site showed no activity, confirming that ss-ds DNA junctions do not themselves act as productive loading sites for Bad. Experiments performed at 30 nM Bad using a substrate with a nick in the top strand (Figure 8B) revealed that the bead never moves further than the distance between the loading site and the nick, and the length distribution is Gaussian-distributed suggesting that translocation is prematurely arrested at the approximate position of the nick (Figure 8C). In complete contrast, experiments using a nick in the bottom strand (Figure 8B) showed similar unwinding length distributions to the MT1 control substrate, being well-fitted by an exponential function and giving a mean value of 870 ± 117 bp (Figure 8D). Together, these data suggest that nicks on the translocating strand strongly inhibit Bad translocation, confirm the 5′-3′ polarity of Bad measured in bulk assays, and strongly suggest that DNA translocation and unwinding are accompanied by simple loop extrusion on the non-translocated strand. This mode of unwinding is also consistent with our observation that the bead is not released when Bad translocation proceeds past the position of the nick on the DNA-nick-bottom substrate.

## DISCUSSION

In this work, we identified and characterised a bacterial helicase–nuclease fusion with primary structure homology to the eukaryotic DNA replication and repair factor DNA2. This enzyme, which we call Bad (bacterial DNA2-like), is rare and sporadically distributed in the bacterial domain. This finding was unexpected given that DNA2 had been considered exclusively eukaryotic in origin (2). We showed here that the biochemical behaviour of Bad is highly similar to that of eukaryotic DNA2 proteins in many respects. Both Bad and DNA2 contain an Fe–S cluster that is important for structural integrity (9). Moreover, they both possess ATPase activity, 5′-3′ ssDNA motor activity, DNA unwinding activity and nuclease activity *in vitro* (35,36), and they both display a preference for binding and/or unwinding substrates with a 5′-flap (37,38). Interestingly, the helicase activity of Bad is autoinhibited by its own nuclease activity (at least *in vitro*) as has also been shown for DNA2. This is presumably because the enzymes cleave DNA ahead of themselves; a counterintuitive activity that could suggest that they act in complex with other proteins (which might overcome this inhibitory effect by providing additional DNA binding sites), or that they are specifically designed to cleave 5′-flap structures until they are sufficiently short to prevent binding. However, the physiological substrate for Bad nuclease activity remains unknown. Cleavage can be observed on circular substrates and therefore appears to be endonucleolytic in nature. This is as expected based on the homology between the N-terminal domain of Bad and RecB-family nucleases which display a Superfamily I endonuclease fold (26). However, our assessment of Bad nuclease activity also unveiled a preference for 5′-overhang substrates, with the positioning of the cleavage sites influenced both by ss–dsDNA junctions and the activity of the DNA motor domain. Nuclease activity was greatly reduced by placing streptavidin blocks at the 5′-end of ssDNA substrates (data not shown), suggesting that endonucleolytic cleavage might be preceded by DNA threading as has been proposed for DNA2 (13).

Single molecule analysis revealed that Bad is a fast and processive DNA motor protein, and that DNA unwinding proceeds by the formation of a ssDNA loop on the non-translocated strand. In our magnetic tweezers set-up, this enables the enzyme to decrease the apparent DNA extension even against high restraining forces. Loop extrusion is an emerging feature of processive DNA helicases and might assist DNA unwinding by disfavouring re-annealing (see (39) for discussion). The bacterial Bad system may provide an interesting model system for studying this activity, particularly using single molecule approaches. Previous analysis of yeast and human Dna2/DNA2 using magnetic tweezers did not provide evidence for ssDNA loop extrusion (12,14). In experiments performed at ∼25 pN, DNA unwinding by Dna2 led to a progressive extension in apparent DNA length as expected for a canonical unwinding activity. This DNA strand separation was also found to be dependent on a single-stranded DNA binding protein (RPA), which was not the case here with Bad (12,40). Finally, observation of Bad activity in the magnetic tweezers set-up is dependent on a ssDNA overhang, as nicks or ssDNA gaps do not act as productive loading sites. Together with the apparent preference for a 5′-flap substrate, this implies again that Bad may preferentially initiate from a free 5′-end perhaps by threading onto the ssDNA.

The physiological role of bacterial Bad proteins is unknown, but they are unlikely to be straightforward orthologues of eukaryotic DNA2 for several reasons. Firstly, the Bad protein is not ubiquitous in bacterial cells, whereas eukaryotic DNA2 is ubiquitous and essential. Furthermore, the major roles played by DNA2 in eukaryotic organisms are apparently already provided by other enzymes in bacteria. For example, the AddAB- and RecBCD-type helicase–nucleases are responsible for DNA end resection to promote homologous recombination (15), and Okazaki fragments are processed by RNaseH (41), although the latter process remains poorly understood in bacteria (42). Finally, Bad proteins all contain a central conserved domain that is not present in any eukaryotic DNA2 protein (Supplementary Figures S1-S3). The structure and function of this domain is completely unknown, as it bears no primary structure homology to anything in the available databases other than uncharacterised Bad proteins. Moreover, domain prediction algorithms also fail to find remote homology with any known domain structures (data not shown). Eukaryotic DNA2 proteins have been found to interact functionally and physically with RecQ-family helicases. However, inspection of the RecQ-family enzymes encoded by bacterial species containing Bad did not reveal any features that obviously differed from canonical RecQ homologues, and which might have hinted at an equivalent interaction in bacteria.

One possible clue as to the cellular role of Bad can be found in the genome organisation of bacteria encoding this protein. In the *Geobacilli*, in all instances we investigated, the *bad* gene was found neighbouring a predicted DNA methyltransferase related to the M subunit of TypeIII restriction enzymes (43,44). Whole genome sequencing of *G. stearothermophilus* 10 (the organism from which the Bad protein studied here originates) using SMRT sequencing suggests that this enzyme methylates the N6-position of adenine in the sequence 5′-GCCAT-3′ (43). Therefore, Bad could be a component of a novel restriction enzyme or any other system which is regulated by DNA methylation. Note however that conventional TypeIII restriction-modification systems do not possess DNA motor activity and are instead ATP-dependent DNA sliding proteins (45). Moreover, even though the TypeI restriction-modification systems do contain *bona fide* DNA motor subunits, these are formed by Superfamily II ‘translocase’ enzymes which move along DNA without unwinding. Therefore, it is plausible that Bad is a subunit of a novel class of restriction enzyme, which might unwind and degrade DNA concomitantly. This hypothesis will be the subject of future work.

## Supplementary Material

gkaa562_Supplemental_File

## References

[1] Symington L.S. Mechanism and regulation of DNA end resection in eukaryotes. Crit. Rev. Biochem. Mol. Biol. 2016; 51:195–212. 27098756 10.3109/10409238.2016.1172552 PMC4957645

[2] Kang Y.H., Lee C.H., Seo Y.S. Dna2 on the road to Okazaki fragment processing and genome stability in eukaryotes. Crit. Rev. Biochem. Mol. Biol. 2010; 45:71–96. 20131965 10.3109/10409230903578593

[3] Budd M.E., Campbell J.L. A yeast replicative helicase, Dna2 helicase, interacts with yeast FEN-1 nuclease in carrying out its essential function. Mol. Cell. Biol. 1997; 17:2136–2142. 9121462 10.1128/mcb.17.4.2136 PMC232061

[4] Budd M.E., Choe W.C., Campbell J.L. DNA2 encodes a DNA helicase essential for replication of eukaryotic chromosomes. J. Biol. Chem. 1995; 270:26766–26769. 7592912 10.1074/jbc.270.45.26766

[5] Zhu Z., Chung W.-H., Shim E.Y., Lee S.E., Ira G. Sgs1 helicase and two nucleases Dna2 and Exo1 resect DNA double-strand break ends. Cell. 2008; 134:981–994. 18805091 10.1016/j.cell.2008.08.037 PMC2662516

[6] Hu J., Sun L., Shen F., Chen Y., Hua Y., Liu Y., Zhang M., Hu Y., Wang Q., Xu W. et al. The intra-S phase checkpoint targets Dna2 to prevent stalled replication forks from reversing. Cell. 2012; 149:1221–132. 22682245 10.1016/j.cell.2012.04.030

[7] Bae S.H., Kim J.A., Choi E., Lee K.H., Kang H.Y., Kim H.D., Kim J.H., Bae K.H., Cho Y., Park C. et al. Tripartite structure of Saccharomyces cerevisiae Dna2 helicase/endonuclease. Nucleic Acids Res. 2001; 29:3069–3079. 11452032 10.1093/nar/29.14.3069 PMC55803

[8] Yeeles J.T., Cammack R., Dillingham M.S. An iron-sulfur cluster is essential for the binding of broken DNA by AddAB-type helicase–nucleases. J. Biol. Chem. 2009; 284:7746–7755. 19129187 10.1074/jbc.M808526200 PMC2658068

[9] Pokharel S., Campbell J.L. Cross talk between the nuclease and helicase activities of Dna2: role of an essential iron-sulfur cluster domain. Nucleic Acids Res. 2012; 40:7821–7830. 22684504 10.1093/nar/gks534 PMC3439918

[10] White M.F., Dillingham M.S. Iron-sulphur clusters in nucleic acid processing enzymes. Curr. Opin. Struct. Biol. 2012; 22:94–100. 22169085 10.1016/j.sbi.2011.11.004

[11] Zhang J., Kasciukovic T., White M.F. The CRISPR associated protein Cas4 is a 5′ to 3′ DNA exonuclease with an iron-sulfur cluster. PLoS One. 2012; 7:e47232. 23056615 10.1371/journal.pone.0047232 PMC3466216

[12] Pinto C., Kasaciunaite K., Seidel R., Cejka P. Human DNA2 possesses a cryptic DNA unwinding activity that functionally integrates with BLM or WRN helicases. Elife. 2016; 5:e18574. 27612385 10.7554/eLife.18574 PMC5030094

[13] Zhou C., Pourmal S., Pavletich N.P. Dna2 nuclease-helicase structure, mechanism and regulation by Rpa. Elife. 2015; 4:e09832. 26491943 10.7554/eLife.09832 PMC4716839

[14] Levikova M., Klaue D., Seidel R., Cejka P. Nuclease activity of Saccharomyces cerevisiae Dna2 inhibits its potent DNA helicase activity. Proc. Natl Acad. Sci. U.S.A. 2013; 110:E1992–E2001. 23671118 10.1073/pnas.1300390110 PMC3670343

[15] Dillingham M.S., Kowalczykowski S.C. RecBCD enzyme and the repair of double-stranded DNA breaks. Microbiol. Mol. Biol. Rev. 2008; 72:642–671. 19052323 10.1128/MMBR.00020-08 PMC2593567

[16] Yeeles J.T., Dillingham M.S. The processing of double-stranded DNA breaks for recombinational repair by helicase–nuclease complexes. DNA Repair (Amst.). 2010; 9:276–285. 20116346 10.1016/j.dnarep.2009.12.016

[17] Chand M.K., Nirwan N., Diffin F.M., van Aelst K., Kulkarni M., Pernstich C., Szczelkun M.D., Saikrishnan K. Translocation-coupled DNA cleavage by the Type ISP restriction-modification enzymes. Nat. Chem. Biol. 2015; 11:870–877. 26389736 10.1038/nchembio.1926 PMC4636054

[18] Dillingham M.S., Tibbles K.L., Hunter J.L., Bell J.C., Kowalczykowski S.C., Webb M.R. Fluorescent single-stranded DNA binding protein as a probe for sensitive, real-time assays of helicase activity. Biophys. J. 2008; 95:3330–3339. 18599625 10.1529/biophysj.108.133512 PMC2547451

[19] Lohman T.M., Green J.M., Beyer R.S. Large-scale overproduction and rapid purification of the Escherichia coli ssb gene product. Expression of the ssb gene under lambda PL control. Biochemistry. 1986; 25:21–25. 3006753 10.1021/bi00349a004

[20] Morris P.D., Tackett A.J., Raney K.D. Biotin-streptavidin-labeled oligonucleotides as probes of helicase mechanisms. Methods. 2001; 23:149–159. 11181034 10.1006/meth.2000.1116

[21] Yeeles J.T., Gwynn E.J., Webb M.R., Dillingham M.S. The AddAB helicase–nuclease catalyses rapid and processive DNA unwinding using a single Superfamily 1A motor domain. Nucleic Acids Res. 2011; 39:2271–2285. 21071401 10.1093/nar/gkq1124 PMC3064778

[22] Matson S.W. Escherichia coli helicase II (urvD gene product) translocates unidirectionally in a 3′ to 5′ direction. J. Biol. Chem. 1986; 261:10169–10175. 2942537

[23] Carrasco C., Gilhooly N.S., Dillingham M.S., Moreno-Herrero F. On the mechanism of recombination hotspot scanning during double-stranded DNA break resection. Proc. Natl Acad. Sci. U.S.A. 2013; 110:E2562–E2571. 23798400 10.1073/pnas.1303035110 PMC3710824

[24] Strick T.R., Allemand J.F., Bensimon D., Croquette V. Behavior of supercoiled DNA. Biophys. J. 1998; 74:2016–2028. 9545060 10.1016/S0006-3495(98)77908-1 PMC1299542

[25] Saikrishnan K., Yeeles J.T., Gilhooly N.S., Krajewski W.W., Dillingham M.S., Wigley D.B. Insights into Chi recognition from the structure of an AddAB-type helicase–nuclease complex. EMBO J. 2012; 31:1568–1578. 22307084 10.1038/emboj.2012.9 PMC3321194

[26] Aravind L., Makarova K.S., Koonin E.V. SURVEY AND SUMMARY: holliday junction resolvases and related nucleases: identification of new families, phyletic distribution and evolutionary trajectories. Nucleic Acids Res. 2000; 28:3417–3432. 10982859 10.1093/nar/28.18.3417 PMC110722

[27] Gilhooly N.S., Gwynn E.J., Dillingham M.S. Superfamily 1 helicases. Front. Biosci. (Schol. Ed.). 2013; 5:206–216. 23277046 10.2741/s367

[28] Singleton M.R., Dillingham M.S., Wigley D.B. Structure and mechanism of helicases and nucleic acid translocases. Annu. Rev. Biochem. 2007; 76:23–50. 17506634 10.1146/annurev.biochem.76.052305.115300

[29] Kaur G., Lewis J.S., van Oijen A.M. Shining a spotlight on DNA: single-molecule methods to visualise DNA. Molecules. 2019; 24:491. 10.3390/molecules24030491 PMC6384704 30704053

[30] Dessinges M.N., Lionnet T., Xi X.G., Bensimon D., Croquette V. Single-molecule assay reveals strand switching and enhanced processivity of UvrD. Proc. Natl Acad. Sci. U.S.A. 2004; 101:6439–6444. 15079074 10.1073/pnas.0306713101 PMC404063

[31] Bustamante C., Bryant Z., Smith S.B. Ten years of tension: single-molecule DNA mechanics. Nature. 2003; 421:423–427. 12540915 10.1038/nature01405

[32] Smith S.B., Cui Y., Bustamante C. Overstretching B-DNA: the elastic response of individual double-stranded and single-stranded DNA molecules. Science. 1996; 271:795–799. 8628994 10.1126/science.271.5250.795

[33] Carrasco C., Pastrana C.L., Aicart-Ramos C., Leuba S.H., Khan S.A., Moreno-Herrero F. Dynamics of DNA nicking and unwinding by the RepC-PcrA complex. Nucleic Acids Res. 2020; 48:2013–2025. 31930301 10.1093/nar/gkz1200 PMC7038956

[34] Lohman T.M., Bjornson K.P. Mechanisms of helicase-catalyzed DNA unwinding. Annu. Rev. Biochem. 1996; 65:169–214. 8811178 10.1146/annurev.bi.65.070196.001125

[35] Kim D.H., Lee K.-H., Kim J.-H., Ryu G.-H., Bae S.-H., Lee B.-C., Moon K.-Y., Byun S.-M., Koo H.-S., Seo Y.-S. Enzymatic properties of the Caenorhabditis elegans Dna2 endonuclease/helicase and a species-specific interaction between RPA and Dna2. Nucleic Acids Res. 2005; 33:1372–1383. 15745997 10.1093/nar/gki255 PMC552954

[36] Masuda-Sasa T., Imamura O., Campbell J.L. Biochemical analysis of human Dna2. Nucleic Acids Res. 2006; 34:1865–1875. 16595800 10.1093/nar/gkl070 PMC1428797

[37] Stewart J.A., Campbell J.L., Bambara R.A. Dna2 is a structure-specific nuclease, with affinity for 5′-flap intermediates. Nucleic Acids Res. 2010; 38:920–930. 19934252 10.1093/nar/gkp1055 PMC2817469

[38] Balakrishnan L., Polaczek P., Pokharel S., Campbell J.L., Bambara R.A. Dna2 exhibits a unique strand end-dependent helicase function. J. Biol. Chem. 2010; 285:38861–38868. 20929864 10.1074/jbc.M110.165191 PMC2998112

[39] Yeeles J.T., van Aelst K., Dillingham M.S., Moreno-Herrero F. Recombination hotspots and single-stranded DNA binding proteins couple DNA translocation to DNA unwinding by the AddAB helicase–nuclease. Mol. Cell. 2011; 42:806–816. 21700225 10.1016/j.molcel.2011.04.012

[40] Levikova M., Pinto C., Cejka P. The motor activity of DNA2 functions as an ssDNA translocase to promote DNA end resection. Genes Dev. 2017; 31:493–502. 28336515 10.1101/gad.295196.116 PMC5393063

[41] Ogawa T., Okazaki T. Function of RNase H in DNA replication revealed by RNase H defective mutants of Escherichia coli. Mol. Gen. Genet. 1984; 193:231–237. 6319961 10.1007/BF00330673

[42] Randall J.R., Nye T.M., Wozniak K.J., Simmons L.A. RNase HIII is important for Okazaki fragment processing in bacillus subtilis. J. Bacteriol. 2019; 201:e00686-18. 30670546 10.1128/JB.00686-18 PMC6416905

[43] Roberts R.J., Vincze T., Posfai J., Macelis D. REBASE–a database for DNA restriction and modification: enzymes, genes and genomes. Nucleic Acids Res. 2015; 43:D298–D299. 25378308 10.1093/nar/gku1046 PMC4383893

[44] Rao D.N., Dryden D.T., Bheemanaik S. Type III restriction-modification enzymes: a historical perspective. Nucleic Acids Res. 2014; 42:45–55. 23863841 10.1093/nar/gkt616 PMC3874151

[45] Szczelkun M.D. Roles for helicases as ATP-Dependent molecular switches. Adv. Exp. Med. Biol. 2013; 767:225–244. 23161014 10.1007/978-1-4614-5037-5_11

