# Supplementary material for: Bulk and single-molecule analysis of a bacterial DNA2-like helicase–nuclease reveals a single-stranded DNA looping motor
Source: Nucleic Acids Res. 2020 Jul 4;48(14):7991–8005. doi: 10.1093/nar/gkaa562 (PMC7430649; doi:10.1093/nar/gkaa562)
Supplement: gkaa562_Supplemental_File [file gkaa562_supplemental_file.pdf]

Bulk and single-molecule analysis of a bacterial DNA2-like helicase nuclease reveals a single-stranded DNA looping motor

Wilkinson, O.J.†[1], Carrasco, C.†[2], Aicart-Ramos, C.[2], Moreno-Herrero, F.*[2] and Dillingham, M.S. *[1]

[1]School of Biochemistry, Biomedical Sciences Building, University of Bristol, University Walk, Bristol, BS8 1TD, UK
[2]Department of Macromolecular Structures, Centro Nacional de Biotecnología, Consejo Superior de Investigaciones Científicas, 28049 Cantoblanco, Madrid, Spain

* To whom correspondence should be addressed. Tel: 0117 3312159; Email: mark.dillingham@bristol.ac.uk. Correspondence may also be addressed to fernando.moreno@cnb.csic.es

† The authors wish it to be known that, in their opinion, the first two authors should be regarded as joint First Authors

SUPPLEMENTARY INFORMATION

Supplementary Methods

Oligonucleotides used in ATPase, translocase and helicase assays.

Name	Sequence
ODN1	CAAGCGGGCGCCTCCCG
ODN2	G(biodT)ACGTATTCAAGATACCTCGTACTCTGTACTGACTGATCCTAGG
ODN3	GTACGTATTCAAGATACCTCGTACTCTGTACTGACTCGGATCC(biodT)A
ODN4	AACGCGCGGGGAGAGGCGGTTTGCGTATTGGGCGCTCTTCCGCTTCCTCGCTCACTGACT
ODN5	CAATACGCAAACCGCCTCTCCCCGCGCGTT
ODN6	AGTCAGTGAGCGAGGAAGCGGAAGAGCGCCCAATACGCAAACCGCCTCTCCCCGCGCGTT
ODN7	AACGCGCGGGGAGAGGCGGTTTGCGTATTG
ODN8	ACTTATCGGTAGTCAGTGAGCGAGGAAGCGGAAGAGCGCCCAATACGCAAACCGCCTCTCCCCGCGCGTT

Assay	Substrate	ODNs
ATPase	17mer ssDNA	1
Translocation	5′-biotin	2
	3′-biotin	3
Helicase	3′-overhang (60)	4+5
	blunt	5+7
	5′-overhang (60)	6+7
	5′-overhang (70)	7+8

DNA substrates for Magnetic Tweezers experiments.

DNA constructs for magnetic tweezers with a 5’-ssDNA overhang or flap sequence (Poly-dT-tail of 37 nt) in a specific-site, consisted of a central fragment ligated to two digoxigenin or biotin-labeled DNA handles. The substrates are based on pNLrep plasmid (kindly gifted by Prof. Dr. Ralf Seidel) that has a DNA sequence which presents five spaced BbvCI restriction sites. The nicking only at one of the two strands using the nicking enzyme Nt.BbvCI results in the formation of short 15–16 bases long fragments that after denaturation creates a 63 b-gap in which desired oligonucleotides can be hybridized (1).

The MT1 DNA substrate was constructed by digesting the pNLrep plasmid with BamHI and BsrGI enzymes (NEB) followed by gel extraction (QIAGEN). The 6610 pb product (Table S2) was then digested with Nt.BbvCI and after inactivation of the enzyme, a 150X excess of Poly-dT oligo (Table S1) was hybridizated into the created gap by heating 5 min at 80°C, and slowly cooling down up to 30°C at a 0.5°C/1 min rate (but increasing 10 sec every minute) in 50 mM Tris pH 8.0 + 1 mM EDTA + 0.1 M NaCl buffer. From the end of the Poly-dT-tail there are 6128 bp up to the bead that can be unwound by the helicase. Handles were PCR-generated from the plasmid pSP73-JY0 (2) or pBlueScript SK+ (Stratagene) (3) using appropriate oligos (Table S1), and adding Bio-dUTP or Dig-dUTP (Roche) respectively, followed by restriction with BsrGI or BamHI. The labeled fragments were then ligated with the central part and the excess of oligonucleotides was removed using two Microspin S-400 columns. DNAs were never exposed to intercalant dyes or UV radiation during their production and were stored at 4°C.

The MT2 DNA substrates (6362 bp) are similar to MT1 DNA but they have a nick in the top (MT2-nick top) or in the bottom strand (MT2-nick bottom) in the region of dsDNA after the Poly-dT that can be unwound by the helicase. To obtain these substrates, a plasmid based in pNLrep plasmid was initially fabricated following a series of clonings to introduce a new BspQI site closer to the Poly-dT-tail in a desired position. This new BspQI site was introduced in both orientations and both new plasmids were checked by DNA sequencing. The MT2 large central parts were generated by enzymatic digestion of these new plasmids with KpnI and PsiI enzymes (Table S2). The purified fragments were treated as described for MT1 substrate. From the end of the Poly-T-tail there are 429 bp up to the nick in the MT2-nick top substrate and 420 bp up to the nick in the MT2-nick bottom substrate that can be unwound by the helicase. Handles were PCR-amplified from the plasmid pSP73-JY0 with appropriate oligos, adding Bio-dUTP or Dig-dUTP, cut with PsiI or KpnI respectively and ligated with the central part. After cleaning with two Microspin S-400 columns, each substrate was digested for 2 h at 50°C with Nt.BspQI nicking enzyme to create the nick, followed by inactivation of the nicking enzyme for 20 min at 80°C.

A control substrate without the flap was prepared in a similar way than MT1 DNA substrate but omitting the steps of digestion with Nt.BbvCI to create a gap for later hybridization of the Poly-dT oligo. Control gap-substrate was prepared in a similar way than MT1 DNA substrate but omitting the steps of hybridization of the Poly-dT oligo after digestion with Nt.BbvCI enzyme.

Supplementary Table S1. DNA oligonucleotides used to construct tweezers substrates

fragment	oligo	sequence
Flap	Poly-dT-overhang	TTTTTTTTTTTTTTTTTTTTTTTTTTTTTTTTTTTTTTTTCAGCTAGCCTCAGCCTACAATCACC
MT1 Bio Handle	FMH-F2-BsrGI	GCGTAAGTTGTACACGACTCACTATAGGGAGACCGGC
	JOE-R1	AGTAAGCGCCGTCAGACCAG
MT1 Dig Handle	PBLUE-F	GACCGAGATAGGGTTGAGTG
	PBLUE-R	CAGGGTCGGAACAGGAGAGC
MT2 Handles	42.FMH_F2_KpnI-PsiI-ScaI	GCGTAAGTGGTACCTTATAAAGTACTCGACTCACTATAGGGAGACCGGC
	JOE-R1	AGTAAGCGCCGTCAGACCAG

Supplementary Table S2. Sequences of DNA fragments used in this work.

- Underlined:63bp-gap created after digestion with the nicking enzyme Nt.BbvCI followed by denaturation.
- Red underlined: where the Poly-dT ssDNA flap is present.
- Black underlined: where the Poly-dT oligo anneals.
- Yellow: base pair in between Nt.BspQI produces a nick in the top strand.
- Blue: base pair in between Nt.BspQI produces a nick in the bottom strand.

Fragment	Size (bp)	Sequence
MT1 central part	6610	GATCCTCAACTGTGAGGAGGCTCACGGACGCGAAGAACAGGCACGCGTGCTGGCAGAAACCCCCGGTATGAC<br>CGTGAAAACGGCCCGCCGCATTCTGGCCGCAGCACCACAGAGTGCACAGGCGCGCAGTGACACTGCGCTGGA<br>TCGTCTGATGCAGGGGGCACCGGCACCGCTGGCTGCAGGTAACCCGGCATCTGATGCCGTTAACGATTTGCT<br>GAACACACCAGTGTAAGGGATGTTTATGACGAGCAAAGAAACCTTTACCCATTACCAGCCGCAGGGCAACAG<br>TGACCCGGCTCATACCGCAACCGCGCCCGGCGGATTGAGTGCGAAAGCGCCTGCAATGACCCCGCTGATGCT<br>GGACACCTCCAGCCGTAAGCTGGTTGCGTGGGATGGCACCACCGACGGTGCTGCCGTTGGCATTCTTGCGGT<br>TGCTGCTCGAGCCTCAGCTCATGTCATCCTCAGCACACTTGACCCTCAGCTCAGCTAGCCTCAGCCTACAAT<br>CACCTCAGCGAATTCGGTGACCCTTACGCGAATCCGCTTTCAGACGTTGACTGGTCGCGTCTGGCAAAAGTT<br>AAAGACCTGACGCCCGGCGAACTGACCGCTGAGTCCTATGACGACAGCTATCTCGATGATGAAGATGCAGAC<br>TGGACTGCGACCGGGCAGGGGCAGAAATCTGCCGGAGATACCAGCTTCACGCTGGCGTGGATGCCCGGAGAG<br>CAGGGGCAGCAGGCGCTGCTGGCGTGGTTTAATGAAGGCGATACCCGTGCCTATAAAATCCGCTTCCCGAAC<br>GGCACGGTCGATGTGTTCCGTGGCTGGGTCAGCAGTATCGGTAAGGCGGTGACGGCGAAGGAAGTGATCACC<br>CGCACGGTGAAAGTCACCAATGTGGGACGTCCGTCGATGGCAGAAGATCGCAGCACGGTAACAGCGGCAACC<br>GGCATGACCGTGACGCCTGCCAGCACCTCGGTGGTGAAAGGGCAGAGCACCACGCTGACCGTGGCCTTCCAG<br>CCGGAGGGCGTAACCGACAAGAGCTTTCGTGCGGTGTCTGCGGATAAAACAAAAGCCACCGTGTCGGTCAGT<br>GGTATGACCATCACCGTGAACGGCGTTGCTGCAGGCAAGGTCAACATTCCGGTTGTATCCGGTAATGGTGAG<br>TTTGCTGCGGTTGCAGAAATTACCGTCACCGCCAGTTAATCCGGAGAGTCAGCGATGTTCCTGAAAACCGAA<br>TCATTTGAACATAACGGTGTGACCGTCACGCTTTCTGAACTGTCAGCCCTGCAGCGCATTGAGCATCTCGCC<br>CTGATGAAACGGCAGGCAGAACAGGCGGAGTCAGACAGCAACCGGAAGTTTACTGTGGAAGACGCCATCAGA<br>ACCGGCGCGTTTCTGGTGGCGATGTCCCTGTGGCATAACCATCCGCAGAAGACGCAGATGCCGTCCATGAAT<br>GAAGCCGTTAAACAGATTGAGCAGGAAGTGCTTACCACCTGGCCCACGGAGGCAATTTCTCATGCTGAAAAC<br>GTGGTGTACCGGCTGTCTGGTATGTATGAGTTTGTGGTGAATAATGCCCCTGAACAGACAGAGGACGCCGGG<br>CCCGCAGAGCCTGTTTCTGCGGGAAAGTGTTCGACGGTGAGCTGAGTTTTGCCCTGAAACTGGCGCGTGAGA<br>TGGGGCGACCCGACTGGCGTGCCATGCTTGCCGGGATGTCATCCACGGAGTATGCCGACTGGCACCGCTTTT<br>ACAGTACCCATTATTTTCATGATGTTCTGCTGGATATGCACTTTTCCGGGCTGACGTACACCGTGCTCAGCC<br>TGTTTTTCAGCGATCCGGATATGCATCCGCTGGATTTCAGTCTGCTGAACCGGCGCGAGGCTGACGAAGAGC<br>CTGAAGATGATGTGCTGATGCAGAAAGCGGCAGGGCTTGCCGGAGGTGTCCGCTTTGGCCCGGACGGGAATG<br>AAGTTATCCCCGCTTCCCCGGATGTGGCGGACATGACGGAGGATGACGTAATGCTGATGACAGTATCAGAAG<br>GGATCGCAGGAGGAGTCCGGTATGGCTGAACCGGTAGGCGATCTGGTCGTTGATTTGAGTCTGGATGCGGCC<br>AGATTTGACGAGCAGATGGCCAGAGTCAGGCGTCATTTTTCTGGTACGGAAAGTGATGCGAAAAAAACAGCG<br>GCAGTCGTTGAACAGTCGCTGAGCCGACAGGCGCTGGCTGCACAGAAAGCGGGGATTTCCGTCGGGCAGTAT<br>AAAGCCGCCATGCGTATGCTGCCTGCACAGTTCACCGACGTGGCCACGCAGCTTGCAGGCGGGCAAAGTCCG<br>TGGCTGATCCTGCTGCAACAGGGGGGGCAGGTGAAGGACTCCTTCGGCGGGATGATCCCCATGTTCAGGGGG<br>CTTGCCGGTGCGATCACCCTGCCGATGGTGGGGGCCACCTCGCTGGCGGTGGCGACCGGTGCGCTGGCGTAT<br>GCCTGGTATCAGGGCAACTCAACCCTGTCCGATTTCAACAAAACGCTGGTCCTTTCCGGCAATCAGGCGGGA<br>CTGACGGCAGATCGTATGCTGGTCCTGTCCAGAGCCGGGCAGGCGGCAGGGCTGACGTTTAACCAGACCAGC<br>GAGTCACTCAGCGCACTGGTTAAGGCGGGGGTAAGCGGTGAGGCTCAGATTGCGTCCATCAGCCAGAGTGTG<br>GCGCGTTTCTCCTCTGCATCCGGCGTGGAGGTGGACAAGGTCGCTGAAGCCTCTAGAGAATGCTACGTACCT<br>GATGAGCTCCAGCTTTTGTTCCCTTTAGTGAGGGTTAATTGCGCGCTTGGCGTAATCATGGTCATAGCTGTT<br>TCCTGTGTGAAATTGTTATCCGCTCACAATTCCACACAACATACGAGCCGGAAGCATAAAGTGTAAAGCCTG<br>GGGTGCCTAATGAGTGAGCTAACTCACATTAATTGCGTTGCGCTCACTGCCCGCTTTCCAGTCGGGAAACCT<br>GTCGTGCCAGCTGCATTAATGAATCGGCCAACGCGCGGGGAGAGGCGGTTTGCGTATTGGGCGCTCTTCCGC<br>TTCCTCGCTCACTGACTCGCTGCGCTCGGTCGTTCGGCTGCGGCGAGCGGTATCAGCTCACTCAAAGGCGGT<br>AATACGGTTATCCACAGAATCAGGGGATAACGCAGGAAAGAACATGTGAGCAAAAGGCCAGCAAAAGGCCAG<br>GAACCGTAAAAAGGCCGCGTTGCTGGCGTTTTTCCATAGGCTCCGCCCCCCTGACGAGCATCACAAAAATCG<br>ACGCTCAAGTCAGAGGTGGCGAAACCCGACAGGACTATAAAGATACCAGGCGTTTCCCCCTGGAAGCTCCCT<br>CGTGCGCTCTCCTGTTCCGACCCTGCCGCTTACCGGATACCTGTCCGCCTTTCTCCCTTCGGGAAGCGTGGC

		GCTTTCTCATAGCTCACGCTGTAGGTATCTCAGTTCGGTGTAGGTCGTTCGCTCCAAGCTGGGCTGTGTGCA<br>CGAACCCCCCGTTCAGCCCGACCGCTGCGCCTTATCCGGTAACTATCGTCTTGAGTCCAACCCGGTAAGACA<br>CGACTTATCGCCACTGGCAGCAGCCACTGGTAACAGGATTAGCAGAGCGAGGTATGTAGGCGGTGCTACAGA<br>GTTCTTGAAGTGGTGGCCTAACTACGGCTACACTAGAAGGACAGTATTTGGTATCTGCGCTCTGCTGAAGCC<br>AGTTACCTTCGGAAAAAGAGTTGGTAGCTCTTGATCCGGCAAACAAACCACCGCTGGTAGCGGTGGTTTTTT<br>TGTTTGCAAGCAGCAGATTACGCGCAGAAAAAAAGGATCTCAAGAAGATCCTTTGATCTTTTCTACGGGGTC<br>TGACGCTCAGTGGAACGAAAACTCACGTTAAGGGATTTTGGTCATGAGATTATCAAAAAGGATCTTCACCTA<br>GATCCTTTTAAATTAAAAATGAAGTTTTAAATCAATCTAAAGTATATATGAGTAAACTTGGTCTGACAGTTA<br>CCAATGCTTAATCAGTGAGGCACCTATCTCAGCGATCTGTCTATTTCGTTCATCCATAGTTGCCTGACTCCC<br>CGTCGTGTAGATAACTACGATACGGGAGGGCTTACCATCTGGCCCCAGTGCTGCAATGATACCGCGAGACCC<br>ACGCTCACCGGCTCCAGATTTATCAGCAATAAACCAGCCAGCCGGAAGGGCCGAGCGCAGAAGTGGTCCTGC<br>AACTTTATCCGCCTCCATCCAGTCTATTAATTGTTGCCGGGAAGCTAGAGTAAGTAGTTCGCCAGTTAATAG<br>TTTGCGCAACGTTGTTGCCATTGCTACAGGCATCGTGGTGTCACGCTCGTCGTTTGGTATGGCTTCATTCAG<br>CTCCGGTTCCCAACGATCAAGGCGAGTTACATGATCCCCCATGTTGTGCAAAAAAGCGGTTAGCTCCTTCGG<br>TCCTCCGATCGTTGTCAGAAGTAAGTTGGCCGCAGTGTTATCACTCATGGTTATGGCAGCACTGCATAATTC<br>TCTTACTGTCATGCCATCCGTAAGATGCTTTTCTGTGACTGGTGAGTACTCAACCAAGTCATTCTGAGAATA<br>GTGTATGCGGCGACCGAGTTGCTCTTGCCCGGCGTCAATACGGGATAATACCGCGCCACATAGCAGAACTTT<br>AAAAGTGCTCATCATTGGAAAACGTTCTTCGGGGCGAAAACTCTCAAGGATCTTACCGCTGTTGAGATCCAG<br>TTCGATGTAACCCACTCGTGCACCCAACTGATCTTCAGCATCTTTTACTTTCACCAGCGTTTCTGGGTGAGC<br>AAAAACAGGAAGGCAAAATGCCGCAAAAAAGGGAATAAGGGCGACACGGAAATGTTGAATACTCATACTCTT<br>CCTTTTTCAATATTATTGAAGCATTTATCAGGGTTATTGTCTCATGAGCGGATACATATTTGAATGTATTTA<br>GAAAAATAAACAAATAGGGGTTCCGCGCACATTTCCCCGAAAAGTGCCACCTAAATTGTAAGCGTTAATATT<br>TTGTTAAAATTCGCGTTAAATTTTTGTTAAATCAGCTCATTTTTTAACCAATAGGCCGAAATCGGCAAAATC<br>CCTTATAAATCAAAAGAATAGACCGAGATAGGGTTGAGTGTTGTTCCAGTTTGGAACAAGAGTCCACTATTA<br>AAGAACGTGGACTCCAACGTCAAAGGGCGAAAAACCGTCTATCAGGGCGATGGCCCACTACGTGAACCATCA<br>CCCTAATCAAGTTTTTTGGGGTCGAGGTGCCGTAAAGCACTAAATCGGAACCCTAAAGGGAGCCCCCGATTT<br>AGAGCTTGACGGGGAAAGCCGGCGAACGTGGCGAGAAAGGAAGGGAAGAAAGCGAAAGGAGCGGGCGCTAGG<br>GCGCTGGCAAGTGTAGCGGTCACGCTGCGCGTAACCACCACACCCGCCGCGCTTAATGCGCCGCTACAGGGC<br>GCGTCCCATTCGCCATTCAGGCTGCGCAACTGTTGGGAAGGGCGATCGGTGCGGGCCTCTTCGCTATTACGC<br>CAGCTGGCGAAAGGGGGATGTGCTGCAAGGCGATTAAGTTGGGTAACGCCAGGGTTTTCCCAGTCACGACGT<br>TGTAAAACGACGGCCAGTGAGCGCGCGTAATACGACTCACTATAGGGCGAATTGGGTACCAGTTCAGGAAGC<br>GGTGATGCTGATAGAAGCCGGACTGAGTACCTACGAGAAAGAGTGCGCAAAACGCGGTGACGACTATCAGGA<br>AATTTTTGCCCAGCAGGTCCGTGAAACGATGGAGCGCCGTGCAGCCGGTCTTAAACCGCCCGCCTGGGCGGC<br>TGCAGCATTTGAATCCGGGCTGCGACAATCAACAGAGGAGGAGAAGAGTGACAGCAGAGCTGCGTAATCTCC<br>CGCATATTGCCAGCATGGCCTTTAATGAGCCGCTGATGCTTGAACCCGCCTATGCGCGGGTTTTCTTTTGTG<br>CGCTTGCAGGCCAGCTTGGGATCAGCAGCCTGACGGATGCGGTGTCCGGCGACAGCCTGACTGCCCAGGAGG<br>CACTCGCGACGCTGGCATTATCCGGTGATGATGACGGACCACGACAGGCCCGCAGTTATCAGGTCATGAACG<br>GCATCGCCGTGCTGCCGGTGTCCGGCACGCTGGTCAGCCGGACGCGGGCGCTGCAGCCGTACTCGGGGATGA<br>CCGGTTACAACGGCATTATCGCCCGTCTGCAACAGGCTGCCAGCGATCCGATGGTGGACGGCATTCTGCTCG<br>ATATGGACACGCCCGGCGGGATGGTGGCGGGGGCATTTGACTGCGCTGACATCATCGCCCGTGTGCGTGACA<br>TAAAACCGGTATGGGCGCTTGCCAACGACATGAACTGCAGTGCAGGTCAGTTGCTTGCCAGTGCCGCCTCCC<br>GGCGTCTGGTCACGCAGACCGCCCGGACAGGCTCCATCGGCGTCATGATGGCTCACAGTAATTACGGTGCTG<br>CGCTGGAGAAACAGGGTGTGGAAATCACGCTGATTTACAGCGGCAGCCATAAGGTGGATGGCAACCCCTACA<br>GCCATCTTCCGGATGACGTCCGGGAGACACTGCAGTCCCGGATGGACGCAACCCGCCAGATGTTTGCGCAGA<br>AGGTGTCGGCATATACCGGCCTGTCCGTGCAGGTTGTGCTGGATACCGAGGCTGCAGT
MT2 DNA-nick top central part	6362	CAGTTCAGGAAGCGGTGATGCTGATAGAAGCCGGACTGAGTACCTACGAGAAAGAGTGCGCAAAACGCGGTG<br>ACGACTATCAGGAAATTTTTGCCCAGCAGGTCCGTGAAACGATGGAGCGCCGTGCAGCCGGTCTTAAACCGC<br>CCGCCTGGGCGGCTGCAGCATTTGAATCCGGGCTGCGACAATCAACAGAGGAGGAGAAGAGTGACAGCAGAG<br>CTGCGTAATCTCCCGCATATTGCCAGCATGGCCTTTAATGAGCCGCTGATGCTTGAACCCGCCTATGCGCGG<br>GTTTTCTTTTGTGCGCTTGCAGGCCAGCTTGGGATCAGCAGCCTGACGGATGCGGTGTCCGGCGACAGCCTG<br>ACTGCCCAGGAGGCACTCGCGACGCTGGCATTATCCGGTGATGATGACGGACCACGACAGGCCCGCAGTTAT<br>CAGGTCATGAACGGCATCGCCGTGCTGCCGGTGTCCGGCACGCTGGTCAGCCGGACGCGGGCGCTGCAGCCG<br>TACTCGGGGATGACCGGTTACAACGGCATTATCGCCCGTCTGCAACAGGCTGCCAGCGATCCGATGGTGGAC<br>GGCATTCTGCTCGATATGGACACGCCCGGCGGGATGGTGGCGGGGGCATTTGACTGCGCTGACATCATCGCC<br>CGTGTGCGTGACATAAAACCGGTATGGGCGCTTGCCAACGACATGAACTGCAGTGCAGGTCAGTTGCTTGCC<br>AGTGCCGCCTCCCGGCGTCTGGTCACGCAGACCGCCCGGACAGGCTCCATCGGCGTCATGATGGCTCACAGT<br>AATTACGGTGCTGCGCTGGAGAAACAGGGTGTGGAAATCACGCTGATTTACAGCGGCAGCCATAAGGTGGAT<br>GGCAACCCCTACAGCCATCTTCCGGATGACGTCCGGGAGACACTGCAGTCCCGGATGGACGCAACCCGCCAG<br>ATGTTTGCGCAGAAGGTGTCGGCATATACCGGCCTGTCCGTGCAGGTTGTGCTGGATACCGAGGCTGCAGTG<br>TACAGCGGTCAGGAGGCCATTGATGCCGGACTGGCTGATGAACTTGTTAACAGCACCGATGCGATCACCGTC<br>ATGCGTGATGCACTGGATGCACGTAAATCCCGTCTCTCAGGAGGGCGAATGACCAAAGAGACTCAATCAACA<br>ACTGTTTCAGCCACTGCTTCGCAGGCTGACGTTACTGACGTGGTGCCAGCGACGGAGGGCGAGAACGCCAGC<br>GCGGCGCAGCCGGACGTGAACGCGCAGATCACCGCAGCGGTTGCGGCAGAAAACAGCCGCATTATGGGGATC<br>CTCAACTGTGAGGAGGCTCACGGACGCGAAGAACAGGCACGCGTGCTGGCAGAAACCCCCGGTATGACCGTG<br>AAAACGGCCCGCCGCATTCTGGCCGCAGCACCACAGAGTGCACAGGCGCGCAGTGACACTGCGCTGGATCGT<br>CTGATGCAGGGGGCACCGGCACCGCTGGCTGCAGGTAACCCGGCATCTGATGCCGTTAACGATTTGCTGAAC<br>ACACCAGTGTAAGGGATGTTTATGACGAGCAAAGAAACCTTTACCCATTACCAGCCGCAGGGCAACAGTGAC<br>CCGGCTCATACCGCAACCGCGCCCGGCGGATTGAGTGCGAAAGCGCCTGCAATGACCCCGCTGATGCTGGAC<br>ACCTCCAGCCGTAAGCTGGTTGCGTGGGATGGCACCACCGACGGTGCTGCCGTTGGCATTCTTGCGGTTGCT<br>GCTCGAGCCTCAGCTCATGTCATCCTCAGCACACTTGACCCTCAGCTCAGCTAGCCTCAGCCTACAATCACC<br>TCAGCGAATTCGGTGACCCTTACGCGAATCCGCTTTCAGACGTTGACTGGTCGCGTCTGGCAAAAGTTAAAG<br>ACCTGACGCCCGGCGAACTGACCGCTGAGTCCTATGACGACAGCTATCTCGATGATGAAGATGCAGACTGGA<br>CTGCGACCGGGCAGGGGCAGAAATCTGCCGGAGATACCAGCTTCACGCTGGCGTGGATGCCCGGAGAGCAGG<br>GGCAGCAGGCGCTGCTGGCGTGGTTTAATGAAGGCGATACCCGTGCCTATAAAATCCGCTTCCCGAACGGCA<br>CGGTCGATGTGTTCCGTGGCTGGGTCAGCAGTATCGGTAAGGCGGTGACGGCGAAGGAAGTGATCACCCGCA<br>CGGTGAAAGTCACCAATGTGGGACGTGGAGTCGACGCTCTTCGTCGACAGGCCGTCGATGGCAGAAGATCGC<br>AGCACGGTAACAGCGGCAACCGGCATGACCGTGACGCCTGCCAGCACCTCGGTGGTGAAAGGGCAGAGCACC<br>ACGCTGACCGTGGCCTTCCAGCCGGAGGGCGTAACCGACAAGAGCTTTCGTGCGGTGTCTGCGGATAAAACA<br>AAAGCCACCGTGTCGGTCAGTGGTATGACCATCACCGTGAACGGCGTTGCTGCAGGCAAGGTCAACATTCCG<br>GTTGTATCCGGTAATGGTGAGTTTGCTGCGGTTGCAGAAATTACCGTCACCGCCAGTTAATCCGGAGAGTCA<br>GCGATGTTCCTGAAAACCGAATCATTTGAACATAACGGTGTGACCGTCACGCTTTCTGAACTGTCAGCCCTG<br>CAGCGCATTGAGCATCTCGCCCTGATGAAACGGCAGGCAGAACAGGCGGAGTCAGACAGCAACCGGAAGTTT<br>ACTGTGGAAGACGCCATCAGAACCGGCGCGTTTCTGGTGGCGATGTCCCTGTGGCATAACCATCCGCAGAAG<br>ACGCAGATGCCGTCCATGAATGAAGCCGTTAAACAGATTGAGCAGGAAGTGCTTACCACCTGGCCCACGGAG<br>GCAATTTCTCATGCTGAAAACGTGGTGTACCGGCTGTCTGGTATGTATGAGTTTGTGGTGAATAATGCCCCT<br>GAACAGACAGAGGACGCCGGGCCCGCAGAGCCTGTTTCTGCGGGAAAGTGTTCGACGGTGAGCTGAGTTTTG<br>CCCTGAAACTGGCGCGTGAGATGGGGCGACCCGACTGGCGTGCCATGCTTGCCGGGATGTCATCCACGGAGT

		ATGCCGACTGGCACCGCTTTTACAGTACCCATTATTTTCATGATGTTCTGCTGGATATGCACTTTTCCGGGC<br>TGACGTACACCGTGCTCAGCCTGTTTTTCAGCGATCCGGATATGCATCCGCTGGATTTCAGTCTGCTGAACC<br>GGCGCGAGGCTGACGTCGACCCTGAAGATGATGTGCTGATGCAGAAAGCGGCAGGGCTTGCCGGAGGTGTCC<br>GCTTTGGCCCGGACGGGAATGAAGTTATCCCCGCTTCCCCGGATGTGGCGGACATGACGGAGGATGACGTAA<br>TGCTGATGACAGTATCAGAAGGGATCGCAGGAGGAGTCCGGTATGGCTGAACCGGTAGGCGATCTGGTCGTT<br>GATTTGAGTCTGGATGCGGCCAGATTTGACGAGCAGATGGCCAGAGTCAGGCGTCATTTTTCTGGTACGGAA<br>AGTGATGCGAAAAAAACAGCGGCAGTCGTTGAACAGTCGCTGAGCCGACAGGCGCTGGCTGCACAGAAAGCG<br>GGGATTTCCGTCGGGCAGTATAAAGCCGCCATGCGTATGCTGCCTGCACAGTTCACCGACGTGGCCACGCAG<br>CTTGCAGGCGGGCAAAGTCCGTGGCTGATCCTGCTGCAACAGGGGGGGCAGGTGAAGGACTCCTTCGGCGGG<br>ATGATCCCCATGTTCAGGGGGCTTGCCGGTGCGATCACCCTGCCGATGGTGGGGGCCACCTCGCTGGCGGTG<br>GCGACCGGTGCGCTGGCGTATGCCTGGTATCAGGGCAACTCAACCCTGTCCGATTTCAACAAAACGCTGGTC<br>CTTTCCGGCAATCAGGCGGGACTGACGGCAGATCGTATGCTGGTCCTGTCCAGAGCCGGGCAGGCGGCAGGG<br>CTGACGTTTAACCAGACCAGCGAGTCACTCAGCGCACTGGTTAAGGCGGGGGTAAGCGGTGAGGCTCAGATT<br>GCGTCCATCAGCCAGAGTGTGGCGCGTTTCTCCTCTGCATCCGGCGTGGAGGTGGACAAGGTCGCTGAAGCC<br>TCTAGAGAATGCTACGTACCTGATGAGCTCCAGCTTTTGTTCCCTTTAGTGAGGGTTAATTGCGCGCTTGGC<br>GTAATCATGGTCATAGCTGTTTCCTGTGTGAAATTGTTATCCGCTCACAATTCCACACAACATACGAGCCGG<br>AAGCATAAAGTGTAAAGCCTGGGGTGCCTAATGAGTGAGCTAACTCACATTAATTGCGTTGCGCTCACTGCC<br>CGCTTTCCAGTCGGGAAACCTGTCGTGCCAGCTGCATTAATGAATCGGCCAACGCGCGGGGAGAGGCGGTTT<br>GCGTATTGGGCGCTCGAGCGCTTCCTCGCTCACTGACTCGCTGCGCTCGGTCGTTCGGCTGCGGCGAGCGGT<br>ATCAGCTCACTCAAAGGCGGTAATACGGTTATCCACAGAATCAGGGGATAACGCAGGAAAGAACATGTGAGC<br>AAAAGGCCAGCAAAAGGCCAGGAACCGTAAAAAGGCCGCGTTGCTGGCGTTTTTCCATAGGCTCCGCCCCCC<br>TGACGAGCATCACAAAAATCGACGCTCAAGTCAGAGGTGGCGAAACCCGACAGGACTATAAAGATACCAGGC<br>GTTTCCCCCTGGAAGCTCCCTCGTGCGCTCTCCTGTTCCGACCCTGCCGCTTACCGGATACCTGTCCGCCTT<br>TCTCCCTTCGGGAAGCGTGGCGCTTTCTCATAGCTCACGCTGTAGGTATCTCAGTTCGGTGTAGGTCGTTCG<br>CTCCAAGCTGGGCTGTGTGCACGAACCCCCCGTTCAGCCCGACCGCTGCGCCTTATCCGGTAACTATCGTCT<br>TGAGTCCAACCCGGTAAGACACGACTTATCGCCACTGGCAGCAGCCACTGGTAACAGGATTAGCAGAGCGAG<br>GTATGTAGGCGGTGCTACAGAGTTCTTGAAGTGGTGGCCTAACTACGGCTACACTAGAAGGACAGTATTTGG<br>TATCTGCGCTCTGCTGAAGCCAGTTACCTTCGGAAAAAGAGTTGGTAGCTCTTGATCCGGCAAACAAACCAC<br>CGCTGGTAGCGGTGGTTTTTTTGTTTGCAAGCAGCAGATTACGCGCAGAAAAAAAGGATCTCAAGAAGATCC<br>TTTGATCTTTTCTACGGGGTCTGACGCTCAGTGGAACGAAAACTCACGTTAAGGGATTTTGGTCATGAGATT<br>ATCAAAAAGGATCTTCACCTAGATCCTTTTAAATTAAAAATGAAGTTTTAAATCAATCTAAAGTATATATGA<br>GTAAACTTGGTCTGACAGTTACCAATGCTTAATCAGTGAGGCACCTATCTCAGCGATCTGTCTATTTCGTTC<br>ATCCATAGTTGCCTGACTCCCCGTCGTGTAGATAACTACGATACGGGAGGGCTTACCATCTGGCCCCAGTGC<br>TGCAATGATACCGCGAGACCCACGCTCACCGGCTCCAGATTTATCAGCAATAAACCAGCCAGCCGGAAGGGC<br>CGAGCGCAGAAGTGGTCCTGCAACTTTATCCGCCTCCATCCAGTCTATTAATTGTTGCCGGGAAGCTAGAGT<br>AAGTAGTTCGCCAGTTAATAGTTTGCGCAACGTTGTTGCCATTGCTACAGGCATCGTGGTGTCACGCTCGTC<br>GTTTGGTATGGCTTCATTCAGCTCCGGTTCCCAACGATCAAGGCGAGTTACATGATCCCCCATGTTGTGCAA<br>AAAAGCGGTTAGCTCCTTCGGTCCTCCGATCGTTGTCAGAAGTAAGTTGGCCGCAGTGTTATCACTCATGGT<br>TATGGCAGCACTGCATAATTCTCTTACTGTCATGCCATCCGTAAGATGCTTTTCTGTGACTGGTGAGTACTC<br>AACCAAGTCATTCTGAGAATAGTGTATGCGGCGACCGAGTTGCTCTTGCCCGGCGTCAATACGGGATAATAC<br>CGCGCCACATAGCAGAACTTTAAAAGTGCTCATCATTGGAAAACGTTCTTCGGGGCGAAAACTCTCAAGGAT<br>CTTACCGCTGTTGAGATCCAGTTCGATGTAACCCACTCGTGCACCCAACTGATCTTCAGCATCTTTTACTTT<br>CACCAGCGTTTCTGGGTGAGCAAAAACAGGAAGGCAAAATGCCGCAAAAAAGGGAATAAGGGCGACACGGAA<br>ATGTTGAATACTCATACTCTTCCTTTTTCAATATTATTGAAGCATTTATCAGGGTTATTGTCTCATGAGCGG<br>ATACATATTTGAATGTATTTAGAAAAATAAACAAATAGGGGTTCCGCGCACATTTCCCCGAAAAGTGCCACC<br>TAAATTGTAAGCGTTAATATTTTGTTAAAATTCGCGTTAAATTTTTGTTAAATCAGCTCATTTTTTAACCAA<br>TAGGCCGAAATCGGCAAAATCCCTTA
MT2 DNA-nick bottom central part	6362	CAGTTCAGGAAGCGGTGATGCTGATAGAAGCCGGACTGAGTACCTACGAGAAAGAGTGCGCAAAACGCGGTG<br>ACGACTATCAGGAAATTTTTGCCCAGCAGGTCCGTGAAACGATGGAGCGCCGTGCAGCCGGTCTTAAACCGC<br>CCGCCTGGGCGGCTGCAGCATTTGAATCCGGGCTGCGACAATCAACAGAGGAGGAGAAGAGTGACAGCAGAG<br>CTGCGTAATCTCCCGCATATTGCCAGCATGGCCTTTAATGAGCCGCTGATGCTTGAACCCGCCTATGCGCGG<br>GTTTTCTTTTGTGCGCTTGCAGGCCAGCTTGGGATCAGCAGCCTGACGGATGCGGTGTCCGGCGACAGCCTG<br>ACTGCCCAGGAGGCACTCGCGACGCTGGCATTATCCGGTGATGATGACGGACCACGACAGGCCCGCAGTTAT<br>CAGGTCATGAACGGCATCGCCGTGCTGCCGGTGTCCGGCACGCTGGTCAGCCGGACGCGGGCGCTGCAGCCG<br>TACTCGGGGATGACCGGTTACAACGGCATTATCGCCCGTCTGCAACAGGCTGCCAGCGATCCGATGGTGGAC<br>GGCATTCTGCTCGATATGGACACGCCCGGCGGGATGGTGGCGGGGGCATTTGACTGCGCTGACATCATCGCC<br>CGTGTGCGTGACATAAAACCGGTATGGGCGCTTGCCAACGACATGAACTGCAGTGCAGGTCAGTTGCTTGCC<br>AGTGCCGCCTCCCGGCGTCTGGTCACGCAGACCGCCCGGACAGGCTCCATCGGCGTCATGATGGCTCACAGT<br>AATTACGGTGCTGCGCTGGAGAAACAGGGTGTGGAAATCACGCTGATTTACAGCGGCAGCCATAAGGTGGAT<br>GGCAACCCCTACAGCCATCTTCCGGATGACGTCCGGGAGACACTGCAGTCCCGGATGGACGCAACCCGCCAG<br>ATGTTTGCGCAGAAGGTGTCGGCATATACCGGCCTGTCCGTGCAGGTTGTGCTGGATACCGAGGCTGCAGTG<br>TACAGCGGTCAGGAGGCCATTGATGCCGGACTGGCTGATGAACTTGTTAACAGCACCGATGCGATCACCGTC<br>ATGCGTGATGCACTGGATGCACGTAAATCCCGTCTCTCAGGAGGGCGAATGACCAAAGAGACTCAATCAACA<br>ACTGTTTCAGCCACTGCTTCGCAGGCTGACGTTACTGACGTGGTGCCAGCGACGGAGGGCGAGAACGCCAGC<br>GCGGCGCAGCCGGACGTGAACGCGCAGATCACCGCAGCGGTTGCGGCAGAAAACAGCCGCATTATGGGGATC<br>CTCAACTGTGAGGAGGCTCACGGACGCGAAGAACAGGCACGCGTGCTGGCAGAAACCCCCGGTATGACCGTG<br>AAAACGGCCCGCCGCATTCTGGCCGCAGCACCACAGAGTGCACAGGCGCGCAGTGACACTGCGCTGGATCGT<br>CTGATGCAGGGGGCACCGGCACCGCTGGCTGCAGGTAACCCGGCATCTGATGCCGTTAACGATTTGCTGAAC<br>ACACCAGTGTAAGGGATGTTTATGACGAGCAAAGAAACCTTTACCCATTACCAGCCGCAGGGCAACAGTGAC<br>CCGGCTCATACCGCAACCGCGCCCGGCGGATTGAGTGCGAAAGCGCCTGCAATGACCCCGCTGATGCTGGAC<br>ACCTCCAGCCGTAAGCTGGTTGCGTGGGATGGCACCACCGACGGTGCTGCCGTTGGCATTCTTGCGGTTGCT<br>GCTCGAGCCTCAGCTCATGTCATCCTCAGCACACTTGACCCTCAGCTCAGCTAGCCTCAGCCTACAATCACC<br>TCAGCGAATTCGGTGACCCTTACGCGAATCCGCTTTCAGACGTTGACTGGTCGCGTCTGGCAAAAGTTAAAG<br>ACCTGACGCCCGGCGAACTGACCGCTGAGTCCTATGACGACAGCTATCTCGATGATGAAGATGCAGACTGGA<br>CTGCGACCGGGCAGGGGCAGAAATCTGCCGGAGATACCAGCTTCACGCTGGCGTGGATGCCCGGAGAGCAGG<br>GGCAGCAGGCGCTGCTGGCGTGGTTTAATGAAGGCGATACCCGTGCCTATAAAATCCGCTTCCCGAACGGCA<br>CGGTCGATGTGTTCCGTGGCTGGGTCAGCAGTATCGGTAAGGCGGTGACGGCGAAGGAAGTGATCACCCGCA<br>CGGTGAAAGTCACCAATGTGGGACGTCCTGTCGACGAAGAGCGTCGACTCCCCGTCGATGGCAGAAGATCGC<br>AGCACGGTAACAGCGGCAACCGGCATGACCGTGACGCCTGCCAGCACCTCGGTGGTGAAAGGGCAGAGCACC<br>ACGCTGACCGTGGCCTTCCAGCCGGAGGGCGTAACCGACAAGAGCTTTCGTGCGGTGTCTGCGGATAAAACA<br>AAAGCCACCGTGTCGGTCAGTGGTATGACCATCACCGTGAACGGCGTTGCTGCAGGCAAGGTCAACATTCCG<br>GTTGTATCCGGTAATGGTGAGTTTGCTGCGGTTGCAGAAATTACCGTCACCGCCAGTTAATCCGGAGAGTCA<br>GCGATGTTCCTGAAAACCGAATCATTTGAACATAACGGTGTGACCGTCACGCTTTCTGAACTGTCAGCCCTG<br>CAGCGCATTGAGCATCTCGCCCTGATGAAACGGCAGGCAGAACAGGCGGAGTCAGACAGCAACCGGAAGTTT<br>ACTGTGGAAGACGCCATCAGAACCGGCGCGTTTCTGGTGGCGATGTCCCTGTGGCATAACCATCCGCAGAAG<br>ACGCAGATGCCGTCCATGAATGAAGCCGTTAAACAGATTGAGCAGGAAGTGCTTACCACCTGGCCCACGGAG<br>GCAATTTCTCATGCTGAAAACGTGGTGTACCGGCTGTCTGGTATGTATGAGTTTGTGGTGAATAATGCCCCT

		GAACAGACAGAGGACGCCGGGCCCGCAGAGCCTGTTTCTGCGGGAAAGTGTTCGACGGTGAGCTGAGTTTTG<br>CCCTGAAACTGGCGCGTGAGATGGGGCGACCCGACTGGCGTGCCATGCTTGCCGGGATGTCATCCACGGAGT<br>ATGCCGACTGGCACCGCTTTTACAGTACCCATTATTTTCATGATGTTCTGCTGGATATGCACTTTTCCGGGC<br>TGACGTACACCGTGCTCAGCCTGTTTTTCAGCGATCCGGATATGCATCCGCTGGATTTCAGTCTGCTGAACC<br>GGCGCGAGGCTGACGTCGACCCTGAAGATGATGTGCTGATGCAGAAAGCGGCAGGGCTTGCCGGAGGTGTCC<br>GCTTTGGCCCGGACGGGAATGAAGTTATCCCCGCTTCCCCGGATGTGGCGGACATGACGGAGGATGACGTAA<br>TGCTGATGACAGTATCAGAAGGGATCGCAGGAGGAGTCCGGTATGGCTGAACCGGTAGGCGATCTGGTCGTT<br>GATTTGAGTCTGGATGCGGCCAGATTTGACGAGCAGATGGCCAGAGTCAGGCGTCATTTTTCTGGTACGGAA<br>AGTGATGCGAAAAAAACAGCGGCAGTCGTTGAACAGTCGCTGAGCCGACAGGCGCTGGCTGCACAGAAAGCG<br>GGGATTTCCGTCGGGCAGTATAAAGCCGCCATGCGTATGCTGCCTGCACAGTTCACCGACGTGGCCACGCAG<br>CTTGCAGGCGGGCAAAGTCCGTGGCTGATCCTGCTGCAACAGGGGGGGCAGGTGAAGGACTCCTTCGGCGGG<br>ATGATCCCCATGTTCAGGGGGCTTGCCGGTGCGATCACCCTGCCGATGGTGGGGGCCACCTCGCTGGCGGTG<br>GCGACCGGTGCGCTGGCGTATGCCTGGTATCAGGGCAACTCAACCCTGTCCGATTTCAACAAAACGCTGGTC<br>CTTTCCGGCAATCAGGCGGGACTGACGGCAGATCGTATGCTGGTCCTGTCCAGAGCCGGGCAGGCGGCAGGG<br>CTGACGTTTAACCAGACCAGCGAGTCACTCAGCGCACTGGTTAAGGCGGGGGTAAGCGGTGAGGCTCAGATT<br>GCGTCCATCAGCCAGAGTGTGGCGCGTTTCTCCTCTGCATCCGGCGTGGAGGTGGACAAGGTCGCTGAAGCC<br>TCTAGAGAATGCTACGTACCTGATGAGCTCCAGCTTTTGTTCCCTTTAGTGAGGGTTAATTGCGCGCTTGGC<br>GTAATCATGGTCATAGCTGTTTCCTGTGTGAAATTGTTATCCGCTCACAATTCCACACAACATACGAGCCGG<br>AAGCATAAAGTGTAAAGCCTGGGGTGCCTAATGAGTGAGCTAACTCACATTAATTGCGTTGCGCTCACTGCC<br>CGCTTTCCAGTCGGGAAACCTGTCGTGCCAGCTGCATTAATGAATCGGCCAACGCGCGGGGAGAGGCGGTTT<br>GCGTATTGGGCGCTCGAGCGCTTCCTCGCTCACTGACTCGCTGCGCTCGGTCGTTCGGCTGCGGCGAGCGGT<br>ATCAGCTCACTCAAAGGCGGTAATACGGTTATCCACAGAATCAGGGGATAACGCAGGAAAGAACATGTGAGC<br>AAAAGGCCAGCAAAAGGCCAGGAACCGTAAAAAGGCCGCGTTGCTGGCGTTTTTCCATAGGCTCCGCCCCCC<br>TGACGAGCATCACAAAAATCGACGCTCAAGTCAGAGGTGGCGAAACCCGACAGGACTATAAAGATACCAGGC<br>GTTTCCCCCTGGAAGCTCCCTCGTGCGCTCTCCTGTTCCGACCCTGCCGCTTACCGGATACCTGTCCGCCTT<br>TCTCCCTTCGGGAAGCGTGGCGCTTTCTCATAGCTCACGCTGTAGGTATCTCAGTTCGGTGTAGGTCGTTCG<br>CTCCAAGCTGGGCTGTGTGCACGAACCCCCCGTTCAGCCCGACCGCTGCGCCTTATCCGGTAACTATCGTCT<br>TGAGTCCAACCCGGTAAGACACGACTTATCGCCACTGGCAGCAGCCACTGGTAACAGGATTAGCAGAGCGAG<br>GTATGTAGGCGGTGCTACAGAGTTCTTGAAGTGGTGGCCTAACTACGGCTACACTAGAAGGACAGTATTTGG<br>TATCTGCGCTCTGCTGAAGCCAGTTACCTTCGGAAAAAGAGTTGGTAGCTCTTGATCCGGCAAACAAACCAC<br>CGCTGGTAGCGGTGGTTTTTTTGTTTGCAAGCAGCAGATTACGCGCAGAAAAAAAGGATCTCAAGAAGATCC<br>TTTGATCTTTTCTACGGGGTCTGACGCTCAGTGGAACGAAAACTCACGTTAAGGGATTTTGGTCATGAGATT<br>ATCAAAAAGGATCTTCACCTAGATCCTTTTAAATTAAAAATGAAGTTTTAAATCAATCTAAAGTATATATGA<br>GTAAACTTGGTCTGACAGTTACCAATGCTTAATCAGTGAGGCACCTATCTCAGCGATCTGTCTATTTCGTTC<br>ATCCATAGTTGCCTGACTCCCCGTCGTGTAGATAACTACGATACGGGAGGGCTTACCATCTGGCCCCAGTGC<br>TGCAATGATACCGCGAGACCCACGCTCACCGGCTCCAGATTTATCAGCAATAAACCAGCCAGCCGGAAGGGC<br>CGAGCGCAGAAGTGGTCCTGCAACTTTATCCGCCTCCATCCAGTCTATTAATTGTTGCCGGGAAGCTAGAGT<br>AAGTAGTTCGCCAGTTAATAGTTTGCGCAACGTTGTTGCCATTGCTACAGGCATCGTGGTGTCACGCTCGTC<br>GTTTGGTATGGCTTCATTCAGCTCCGGTTCCCAACGATCAAGGCGAGTTACATGATCCCCCATGTTGTGCAA<br>AAAAGCGGTTAGCTCCTTCGGTCCTCCGATCGTTGTCAGAAGTAAGTTGGCCGCAGTGTTATCACTCATGGT<br>TATGGCAGCACTGCATAATTCTCTTACTGTCATGCCATCCGTAAGATGCTTTTCTGTGACTGGTGAGTACTC<br>AACCAAGTCATTCTGAGAATAGTGTATGCGGCGACCGAGTTGCTCTTGCCCGGCGTCAATACGGGATAATAC<br>CGCGCCACATAGCAGAACTTTAAAAGTGCTCATCATTGGAAAACGTTCTTCGGGGCGAAAACTCTCAAGGAT<br>CTTACCGCTGTTGAGATCCAGTTCGATGTAACCCACTCGTGCACCCAACTGATCTTCAGCATCTTTTACTTT<br>CACCAGCGTTTCTGGGTGAGCAAAAACAGGAAGGCAAAATGCCGCAAAAAAGGGAATAAGGGCGACACGGAA<br>ATGTTGAATACTCATACTCTTCCTTTTTCAATATTATTGAAGCATTTATCAGGGTTATTGTCTCATGAGCGG<br>ATACATATTTGAATGTATTTAGAAAAATAAACAAATAGGGGTTCCGCGCACATTTCCCCGAAAAGTGCCACC<br>TAAATTGTAAGCGTTAATATTTTGTTAAAATTCGCGTTAAATTTTTGTTAAATCAGCTCATTTTTTAACCAA<br>TAGGCCGAAATCGGCAAAATCCCTTA

Supplementary References

1. Luzzietti N, Brutzer H, Klaue D, Schwarz FW, Staroske W, Clausing S, et al. Efficient preparation of internally modified single-molecule constructs using nicking enzymes. Nucleic Acids Res. 2011;39(3):e15.
2. Fili N, Mashanov GI, Toseland CP, Batters C, Wallace MI, Yeeles JT, et al. Visualizing helicases unwinding DNA at the single molecule level. Nucleic Acids Res. 2010;38(13):4448-57.
3. Pastrana CL, Carrasco C, Akhtar P, Leuba SH, Khan SA, Moreno-Herrero F. Force and twist dependence of RepC nicking activity on torsionally-constrained DNA molecules. Nucleic Acids Res. 2016;44(18):8885-96.
4. Crooks GE, Hon G, Chandonia JM, Brenner SE. WebLogo: a sequence logo generator. Genome Res. 2004;14(6):1188-90.
5. Aravind L, Makarova KS, Koonin EV. SURVEY AND SUMMARY: holliday junction resolvases and related nucleases: identification of new families, phyletic distribution and evolutionary trajectories. Nucleic Acids Res. 2000;28(18):3417-32.
6. Gilhooly NS, Gwynn EJ, Dillingham MS. Superfamily 1 helicases. Front Biosci (Schol Ed). 2013;5:206-16.
7. Chen C, Natale DA, Finn RD, Huang H, Zhang J, Wu CH, et al. Representative proteomes: a stable, scalable and unbiased proteome set for sequence analysis and functional annotation. PLoS One. 2011;6(4):e18910.
8. Papadopoulos JS, Agarwala R. COBALT: constraint-based alignment tool for multiple protein sequences. Bioinformatics. 2007;23(9):1073-9.
9. Saikrishnan K, Yeeles JT, Gilhooly NS, Krajewski WW, Dillingham MS, Wigley DB. Insights into Chi recognition from the structure of an AddAB-type helicase-nuclease complex. EMBO J. 2012;31(6):1568-78.
10. Zhou C, Pourmal S, Pavletich NP. Dna2 nuclease-helicase structure, mechanism and regulation by Rpa. Elife. 2015;4.

Supplementary Figures

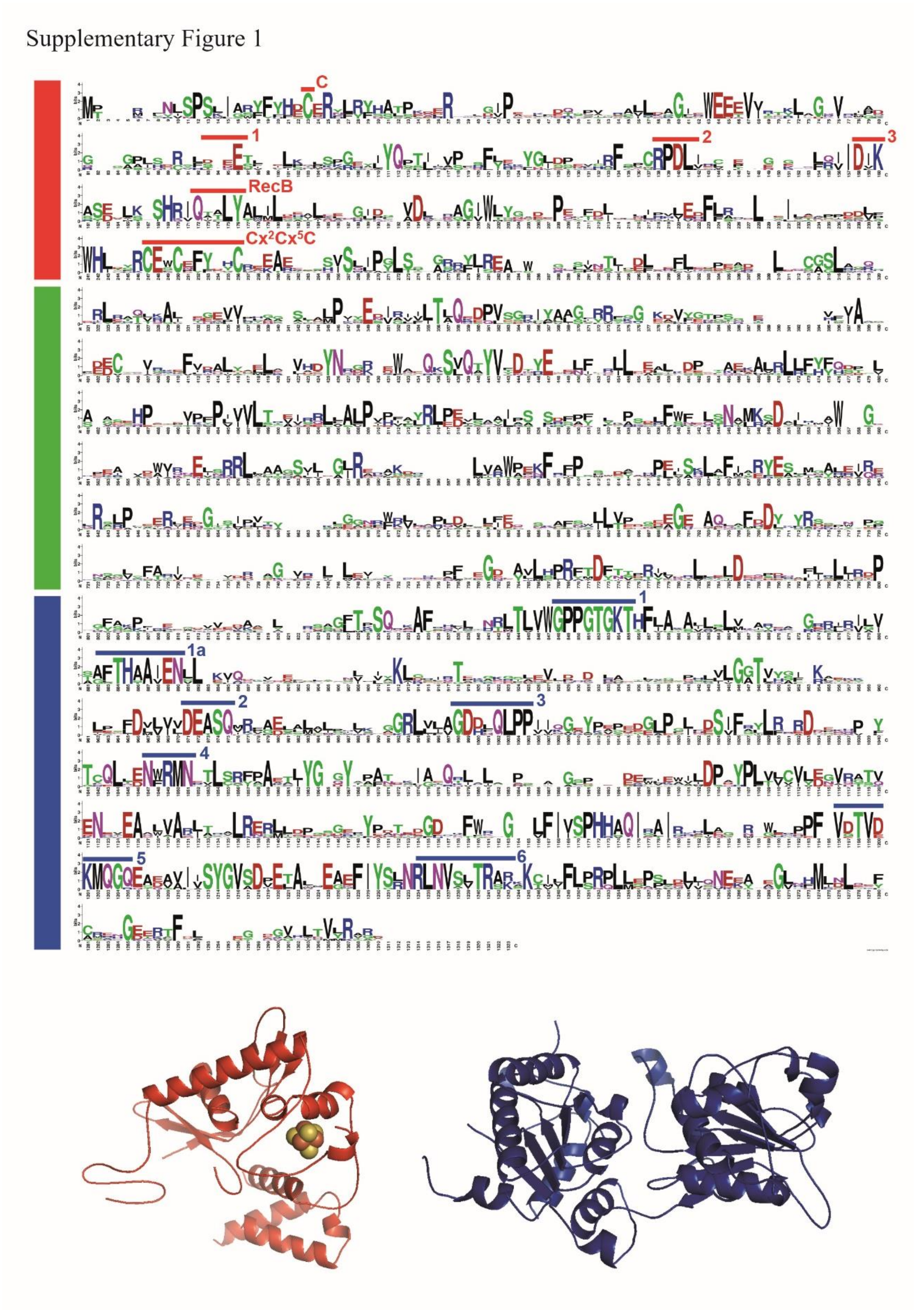

Supplementary Figure 1 *Domain structure of bacterial DNA2-like (Bad) proteins.* (Top) Multiple sequence alignment of bacterial and archaeal DNA2-like proteins shown in WebLogo format (4). The bars to the left of the logo approximately delineate the overall architecture showing the N-terminal Fe-S associated RecB-family nuclease domain (red), a central domain (green) and a C-terminal

Superfamily 1B helicase domain (blue). Aside from a short region of predicted coiled-coil, no information can be gleaned from bioinformatics approaches about the likely structure or function of the central green domain. Amino acid motifs that are characteristic of the Fe-S RecB-family nuclease are highlighted with red bars above the logo and include the flanking cysteine residues, nuclease motifs 1-3, and the RecB-family specific motif (5). Amino acid motifs 1-6 that are characteristic of SF1B helicases are highlighted with blue bars above the logo (6). The multiple sequence alignment was created by BLAST-based homology searching using a representative proteome database (7), followed by manual editing of the resulting alignment in COBALT (8) to remove sequences of significantly different length or those which did not conform to the three domain architecture indicated above. The sequences used for the final multiple sequence alignment were the Bad proteins from the following bacterial and archaeal species: *Geobacillus thermoleovorans* (WP_011230911.1), *Parageobacillus toebii* (WP_062677896.1), *Anoxybacillus flavithermus* (WK1 ACJ33552.1), *Methanohalobium evestigatum* (WP_013193644.1), *Methanosphaerula palustris* (WP_012618149.1), *Methanospirillum hungatei* (WP_011448513.1), *Methanoculleus marisnigri* (WP_011844558.1), *Methanoculleus marisnigri* (KUL02919.1), *Methanoculleus bourgensis* (WP_014866635.1), *Methanofollis liminatans* (WP_004038903.1), *Candidatus Competibacter denitrificans* (WP_048677101.1). (Bottom) Modelled structures of the N-terminal Fe-S nuclease (red) and C-terminal helicase (blue) domains of *G. stearothermophilus* Bad. The models were generated using Phyre2 and are based on homology to *B. subtilis* AddB (PDB code: 3U44; (9)) and mouse DNA2 (PDB code: 5EAX; (10)) respectively.

Supplementary Figure 2

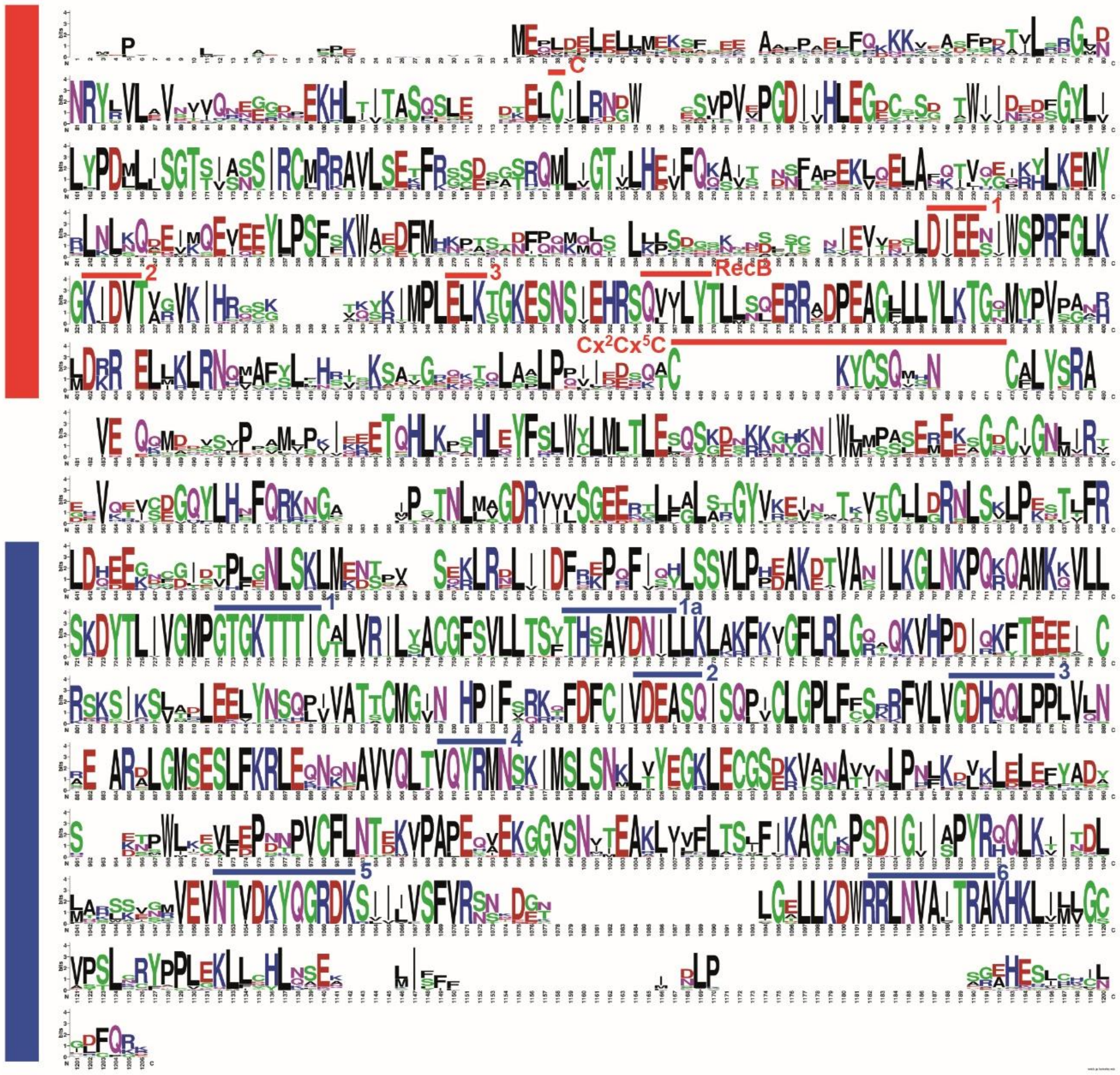

Supplementary Figure 2 *Domain structure of DNA2 from human and related species.* Multiple sequence alignment of higher eukaryotic DNA2 proteins shown in WebLogo format (4). The bars to the left of the logo approximately delineate the overall architecture showing the N-terminal Fe-S associated RecB-family nuclease domain (red) and a C-terminal Superfamily 1B helicase domain (blue). Amino acid motifs that are characteristic of the nuclease and helicase domains are labelled as in **Supplementary Figure 1**. The multiple sequence alignment was created by BLAST-based homology searching using mouse DNA2 in a representative proteome database (7), followed by manual editing of the resulting alignment in COBALT (8) to remove sequences of significantly different length or those which did not conform to the domain architecture indicated above.

Supplementary Figure 3

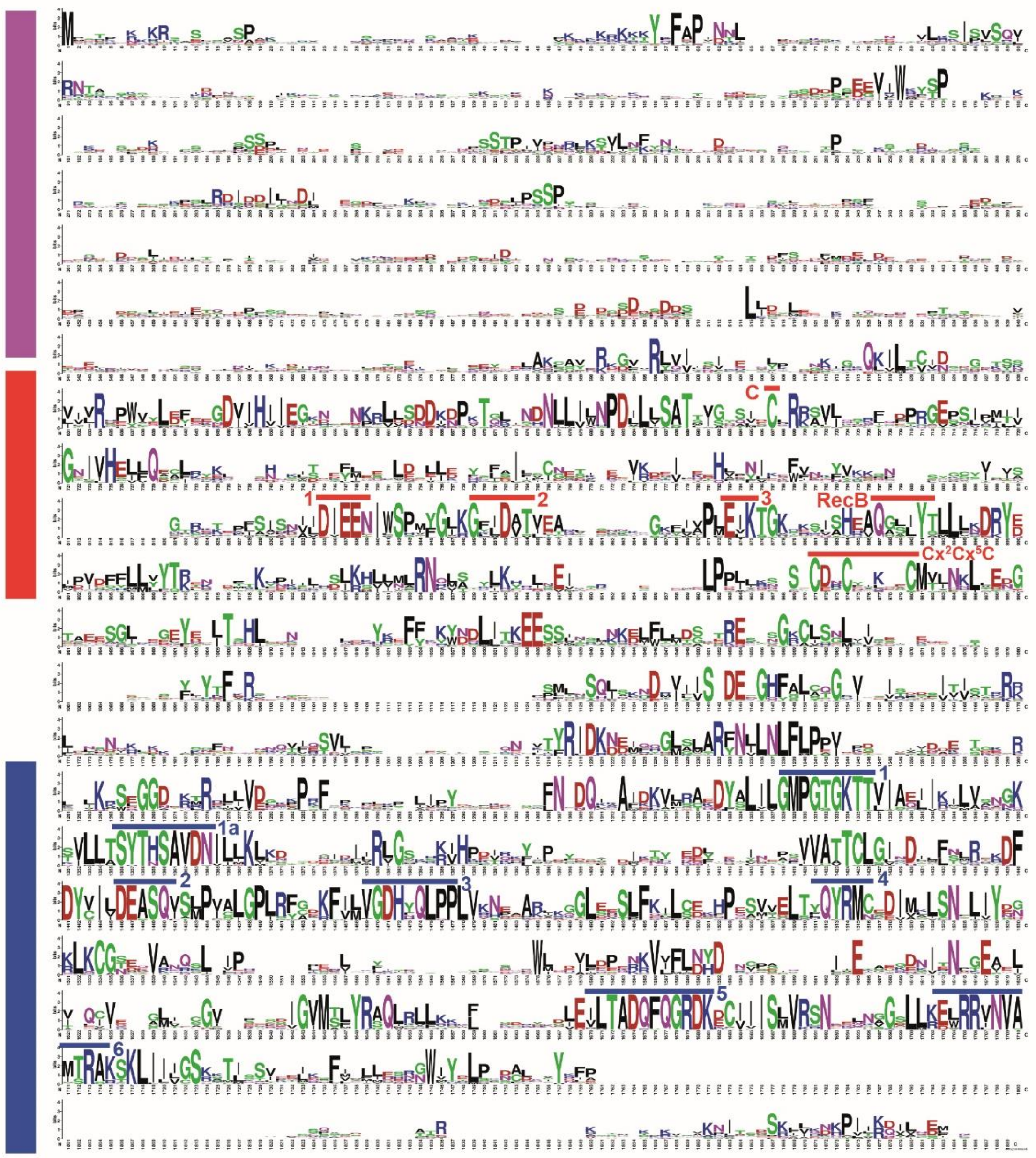

Supplementary Figure 3 *Domain structure of yeast Dna2 proteins.* Multiple sequence alignment of *S. cerevisiae* and closely-related DNA2 proteins shown in WebLogo format (4). The bars to the left of the logo approximately delineate the overall architecture showing the N-terminal yeast specific domain (purple), the Fe-S associated RecB-family nuclease domain (red) and a C-terminal Superfamily 1B helicase domain (blue). Amino acid motifs that are characteristic of the nuclease and helicase domains are labelled as in **Supplementary Figure 1**. The multiple sequence alignment was created by BLAST-based homology searching using *S. cerevisiae* Dna2 in a representative proteome database (7), followed by manual editing of the resulting alignment in COBALT (8) to remove sequences of significantly different length or those which did not conform to the domain architecture indicated above.

Supplementary Figure 4

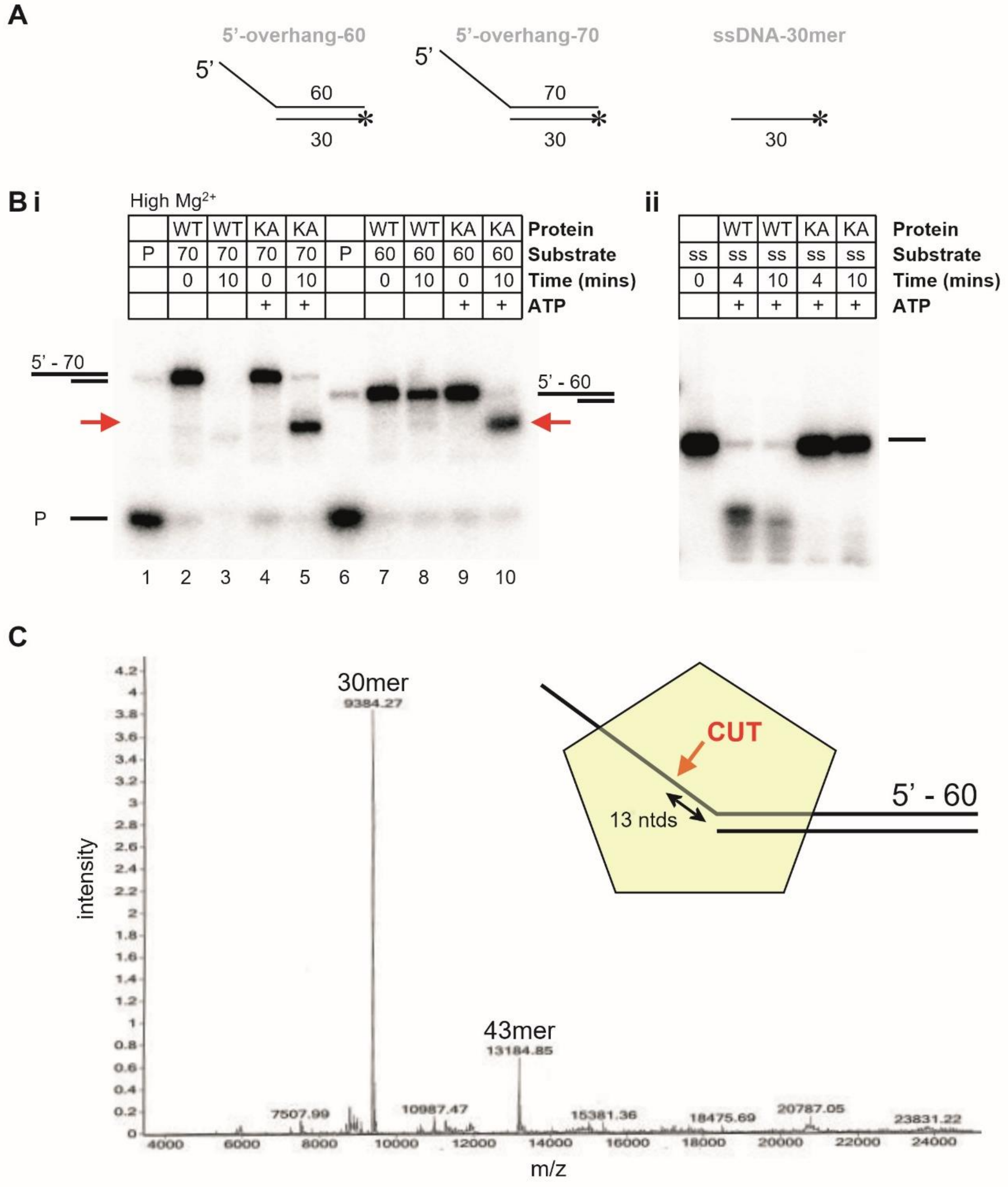

Supplementary Figure 4 *Bad binds and cleaves a 5'-ssDNA overhang at a precise location relative to the ss-dsDNA junction in an ATP-dependent manner.* (A) Schematic of the DNA substrates used in these assays. The sequences are available in the Supplementary Methods. (B) (i) Coupled helicase-nuclease assays were performed with wild type or mutant Bad protein in high free Mg^{2+} ion conditions and with or without ATP. In order to distinguish between cleavage of DNA relative to the 5'-end of the long strand or to the ss-dsDNA junction two DNA substrates were used with different length top strands of 60 or 70 ntds. P is a marker DNA for the position of the unwound short DNA strand. (ii) Nuclease assays were performed with a 30mer ssDNA oligonucleotide (1 nM) and wild type or mutant Bad protein (5 nM) in high free Mg^{2+} ion conditions with ATP. In the absence of DNA motor activity (KA mutant), no

degradation of DNA is observed showing that the active nuclease domain does not display exonuclease activity. See main text for discussion. (C) Schematic showing the Bad cleavage position on the 5’-60 DNA as revealed by mass spectrometric analysis of the products. The analysis revealed oligonucleotides with the exact mass of the short ssDNA strand (9384.1 Da), as well as the long DNA strand that had been cut precisely at a position 13 nucleotides from the junction to leave a 5’-phosphate (red arrow; 13183.5 Da). The 5’-portion of the long DNA strand was not detected in this analysis suggesting it may have been degraded.

Supplementary Figure 5

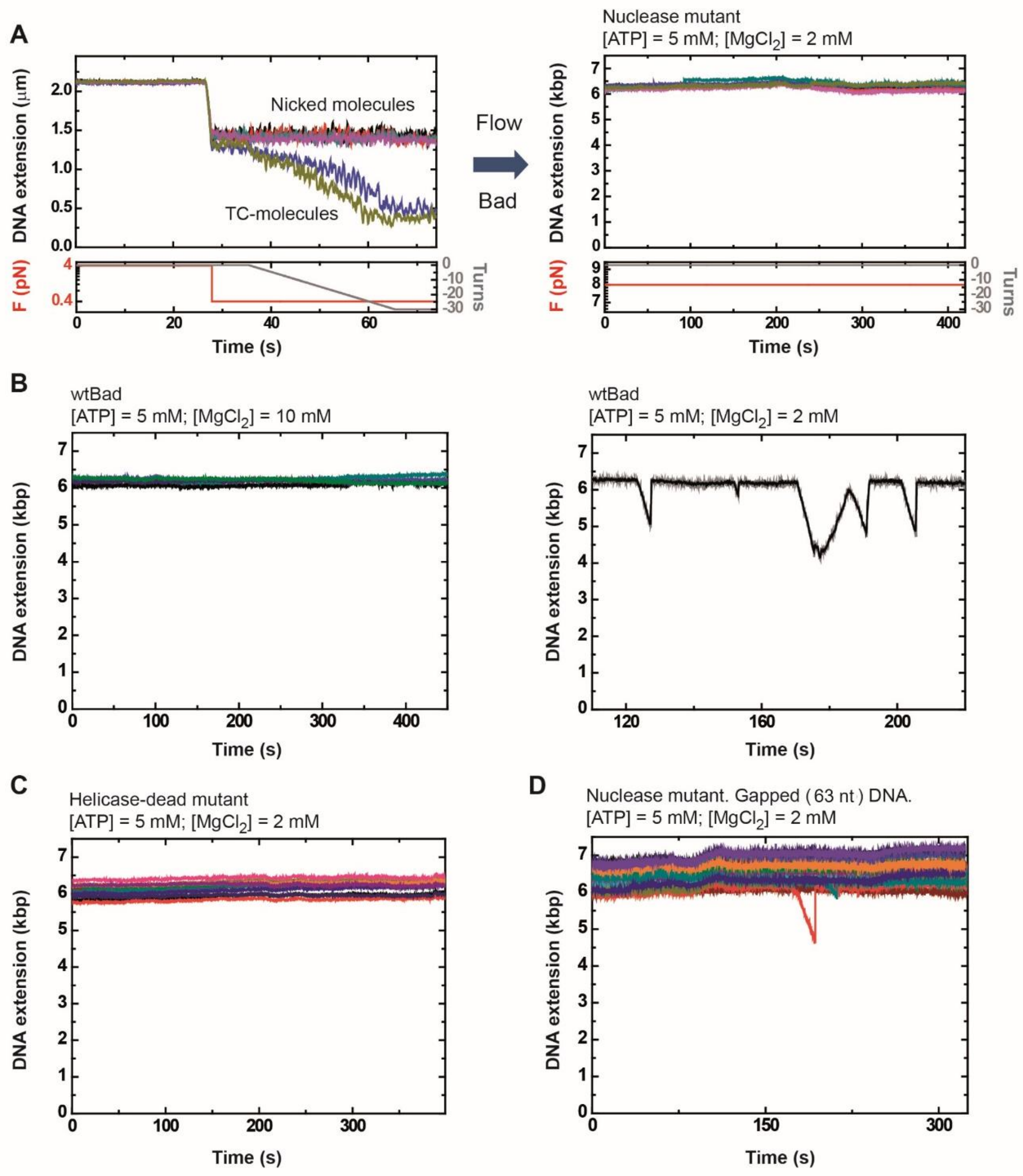

Supplementary Figure 5. *Single molecule control experiments.* (A) Activity of nuclease-dead Bad mutant on torsionally-constrained (N = 2) and nicked (N = 4) DNA molecules. No unwinding events were observed. (B) Wild type Bad activity at high or low free Mg^{2+} conditions as indicated. Unwinding events were only observed under low free Mg^{2+} conditions in which the nuclease activity is suppressed. (C) Helicase-dead Bad mutant under low Mg^{2+} conditions. N = 13, no unwinding events were observed. (D) Nuclease-dead Bad mutant activity on DNA containing a 63 base gap. N = 34, 3 short unwinding and 1 backslide events were detected.

Supplementary Figure 6

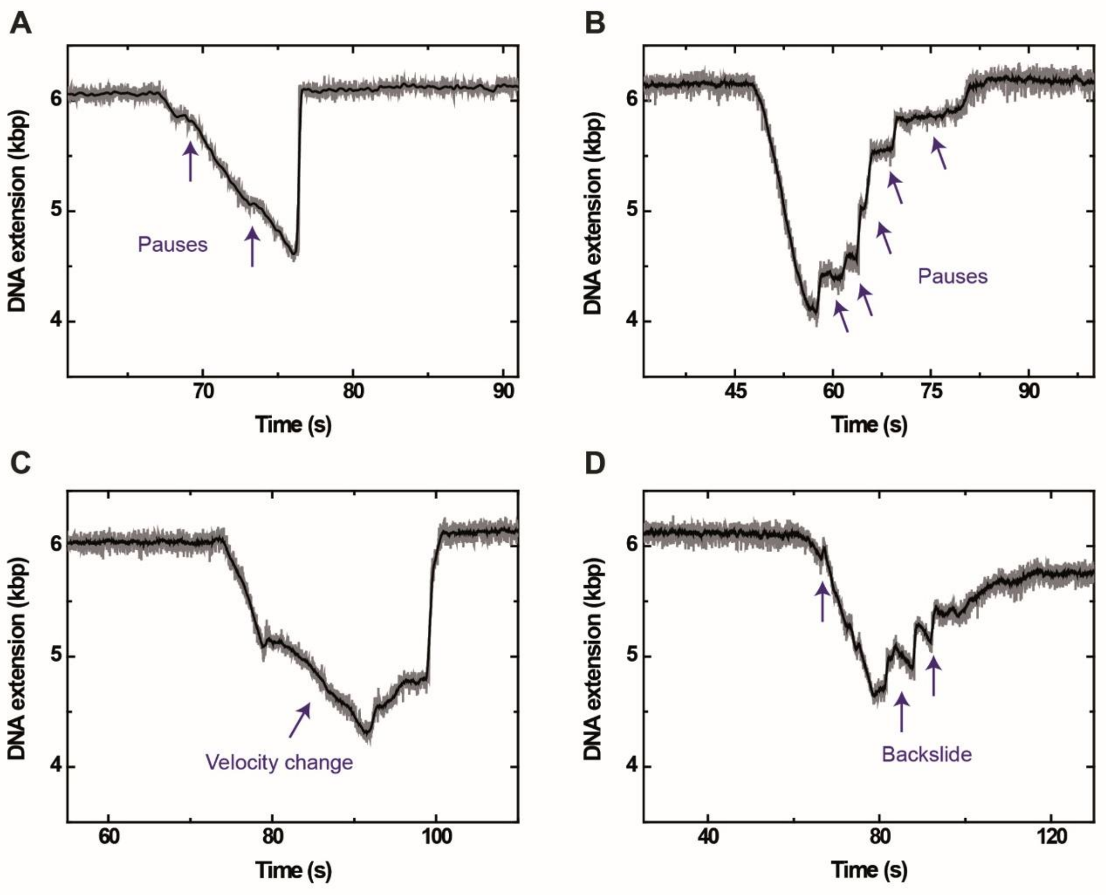

Supplementary Figure 6. *Examples of pauses, velocity changes and backsliding during Bad translocation.* (A) Pauses during unwinding. (B) Pauses during rehybridization. (C) Velocity changes during unwinding. (D) Backsliding (rapid rehybridization following by restart of unwinding). Experiments were performed with 163 nM Bad under the low free magnesium ion conditions. Quantification of these events for a range of Bad concentrations is shown in the main text **Table 1**.

Supplementary Figure 7

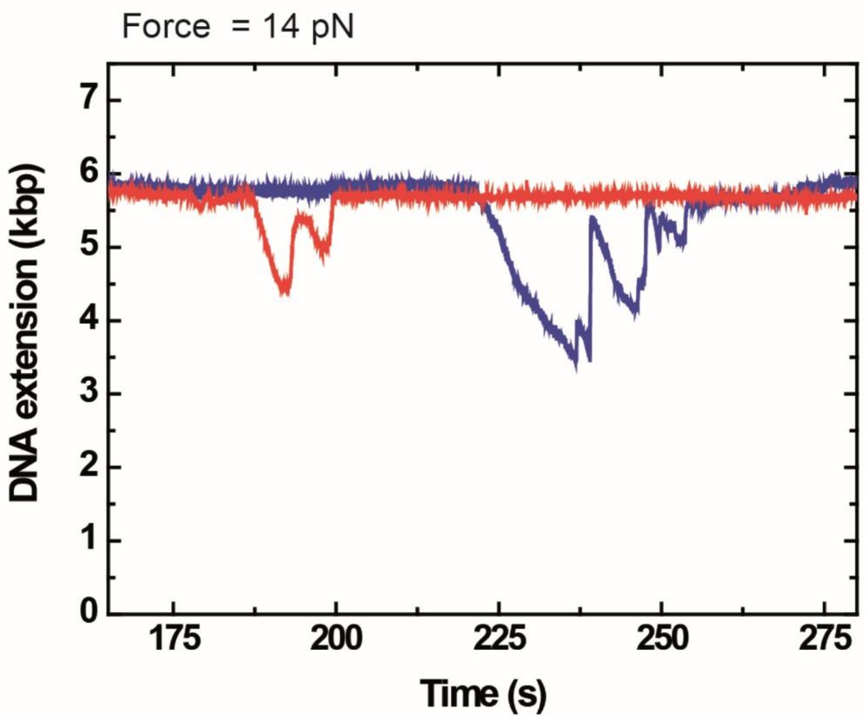

Supplementary Figure 7. *Examples of DNA unwinding traces at 14 pN applied force.* Unwinding traces collected at high (14 pN) force still exhibit Bad-dependent decreases in the bead height.

Supplementary Figure 8

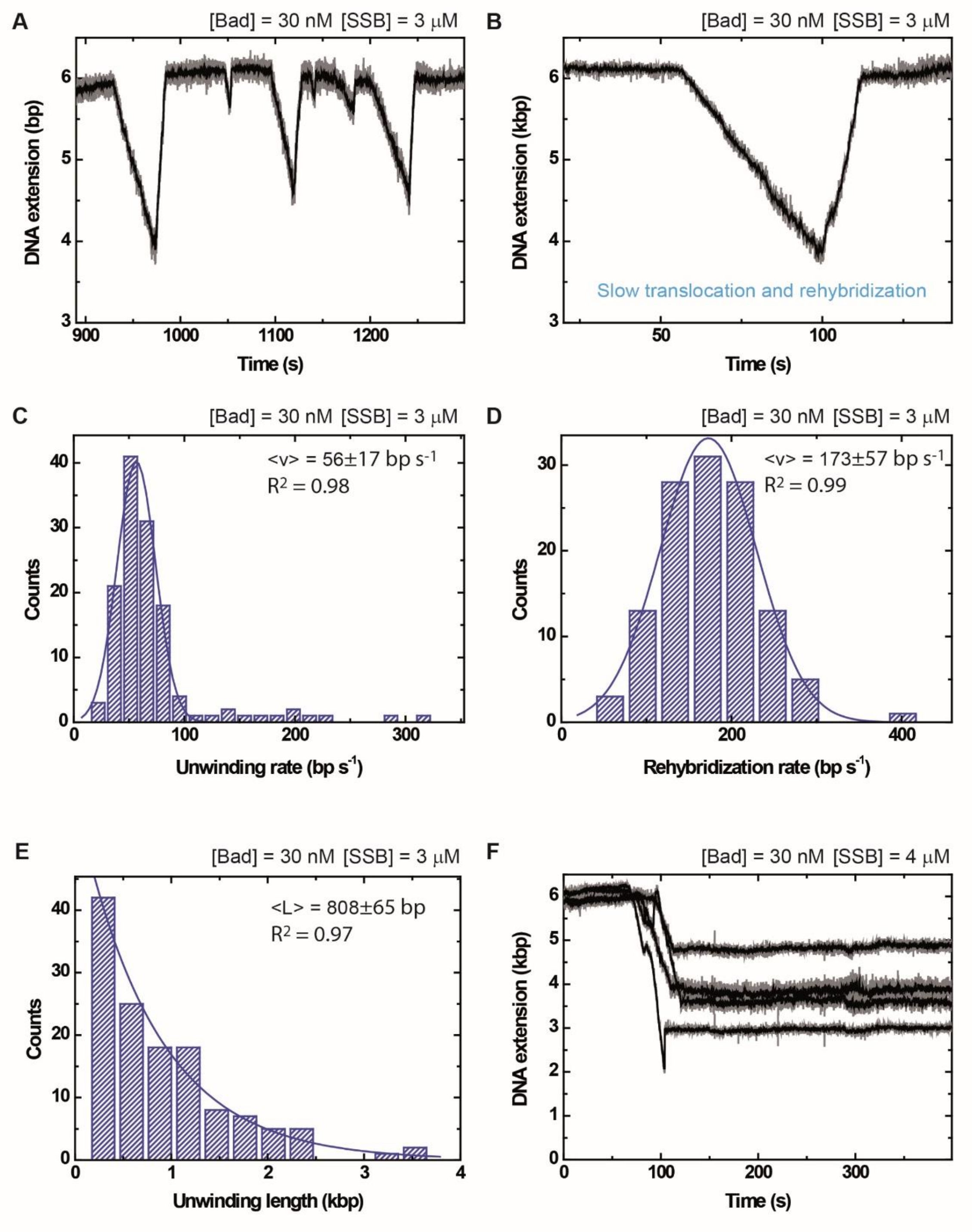

Supplementary Figure 8. *DNA unwinding and rehybridization in the presence of SSB.* Examples of unwinding and rehybridization events are shown in (A) and (B) for the nuclease-dead mutant in the presence of 3 µM SSB and 30 nM Bad. We observed a much slower rehybridization than in the absence of SSB. (C) Unwinding rate distribution. Mean unwinding rate was 56±17 bp s^{-1} (SD, n = 131). (D) Rehybridization rate distribution. Mean rehybridization rate was 173±57 bp s^{-1} (SD, n = 131). Both distributions were well fitted with a Gaussian function (blue lines). (E) Unwinding length distribution. Mean unwinding length was <L> = 808 ± 65 bp (error of fitting, n = 131). The mean unwinding length in

the presence of SSB is smaller than that obtained without SSB proteins (see Figure 6). Interestingly, the percentage of backsliding events in the data was reduced by 10 % in the presence of SSB. A total of 36 tethered beads were analysed showing a total of 131 unwinding events. (F) At 4 µM SSB, complete rehybridization events were never observed, although partial and short rehybridization events did still occur.
